# Metabolomics Simultaneously Derives Benchmark Dose Estimates and Discovers Metabolic Biotransformations in a Rat Bioassay

**DOI:** 10.1021/acs.chemrestox.4c00002

**Published:** 2024-06-06

**Authors:** Elena Sostare, Tara J. Bowen, Thomas N. Lawson, Anne Freier, Xiaojing Li, Gavin R. Lloyd, Lukáš Najdekr, Andris Jankevics, Thomas Smith, Dorsa Varshavi, Christian Ludwig, John K. Colbourne, Ralf J. M. Weber, David M. Crizer, Scott S. Auerbach, John R. Bucher, Mark R. Viant

**Affiliations:** † Michabo Health Science Ltd., Union House, 111 New Union Street, Coventry CV1 2NT, U.K.; ‡ School of Biosciences, University of Birmingham, Birmingham B15 2TT, U.K.; § Phenome Centre Birmingham, University of Birmingham, Birmingham B15 2TT, U.K.; ∥ Division of Translational Toxicology, National Institute of Environmental Health Sciences, Research Triangle Park NC 27709, North Carolina, United States

## Abstract

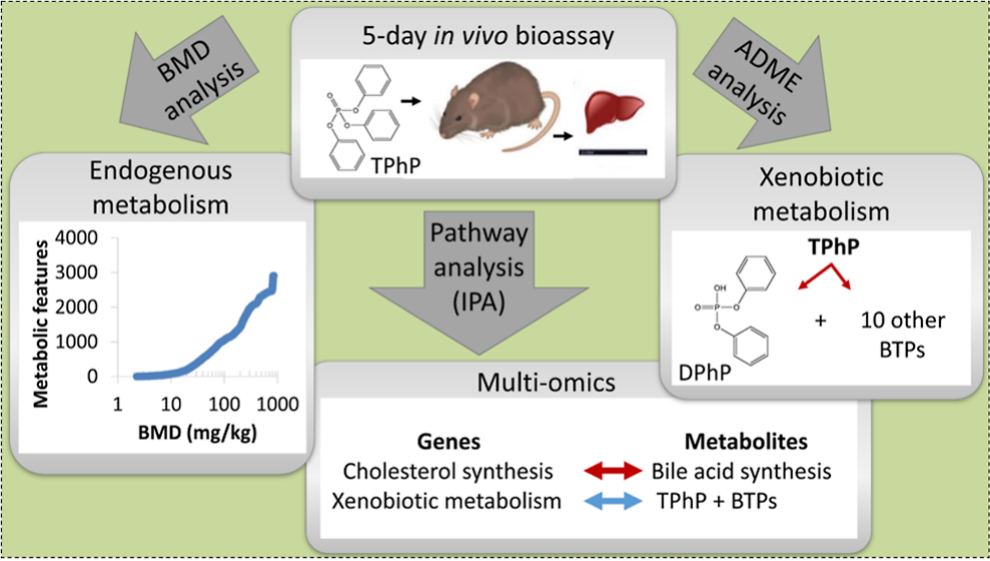

Benchmark dose (BMD) modeling estimates the dose of a chemical that causes a perturbation from baseline. Transcriptional BMDs have been shown to be relatively consistent with apical end point BMDs, opening the door to using molecular BMDs to derive human health-based guidance values for chemical exposure. Metabolomics measures the responses of small-molecule endogenous metabolites to chemical exposure, complementing transcriptomics by characterizing downstream molecular phenotypes that are more closely associated with apical end points. The aim of this study was to apply BMD modeling to in vivo metabolomics data, to compare metabolic BMDs to both transcriptional and apical end point BMDs. This builds upon our previous application of transcriptomics and BMD modeling to a 5-day rat study of triphenyl phosphate (TPhP), applying metabolomics to the same archived tissues. Specifically, liver from rats exposed to five doses of TPhP was investigated using liquid chromatography–mass spectrometry and ^1^H nuclear magnetic resonance spectroscopy-based metabolomics. Following the application of BMDExpress2 software, 2903 endogenous metabolic features yielded viable dose-response models, confirming a perturbation to the liver metabolome. Metabolic BMD estimates were similarly sensitive to transcriptional BMDs, and more sensitive than both clinical chemistry and apical end point BMDs. Pathway analysis of the multiomics data sets revealed a major effect of TPhP exposure on cholesterol (and downstream) pathways, consistent with clinical chemistry measurements. Additionally, the transcriptomics data indicated that TPhP activated xenobiotic metabolism pathways, which was confirmed by using the underexploited capability of metabolomics to detect xenobiotic-related compounds. Eleven biotransformation products of TPhP were discovered, and their levels were highly correlated with multiple xenobiotic metabolism genes. This work provides a case study showing how metabolomics and transcriptomics can estimate mechanistically anchored points-of-departure. Furthermore, the study demonstrates how metabolomics can also discover biotransformation products, which could be of value within a regulatory setting, for example, as an enhancement of OECD Test Guideline 417 (toxicokinetics).

## 1 Introduction

In determining the human and environmental health risks associated with industrial chemicals, benchmark dose (BMD) modeling has become a valuable quantitative approach to estimate a point of departure (PoD) from baseline,^1,2^ that is, the dose corresponding to the threshold of toxicity that serves as an important metric for regulatory guidance on chemical safety.^3^ In general, BMD modeling describes the process of fitting mathematical models to experimental toxicity data and deriving the BMD at a predefined benchmark response (BMR) level, often 10% deviation from the baseline in the case of dichotomous data or 1 standard deviation in the case of continuous data when a predefined level change associated with toxicity is not known.^4^ The lower 95% confidence limit of the BMD (BMDL) is commonly used to derive a more conservative limit for human risk guidance,^5^ offering several advantages over no-observed-adverse-effect levels (NOAELs),^6,7^ including independence from the actual dose levels in a study, improved efficiency when investigating small sample sizes, and incorporation of the entire dose–response curve.^3,8^ While often applied to apical end points (or adverse outcomes) such as organ weight, BMD modeling of transcriptomics data have also become routine.^2,9−11^ In particular, transcriptional BMD values have been shown to be relatively consistent with apical end point BMDs,^12−15^ opening the possibility of using a molecular BMD to derive a health-based guidance value for chemical exposure. Recently, the Division of Translational Toxicology (DTT), U.S. Department of Health and Human Services, evaluated the applicability of transcriptomics and BMD modeling to a 5 -day in vivo (rat) study to derive molecular BMDs and to contrast these with the responses of apical end points.^16,17^

Metabolomic technologies can be used to investigate the responses of large numbers of small-molecule endogenous metabolites to chemical exposure, offering a complementary approach to transcriptomics by characterizing downstream molecular phenotypes that are more closely associated with apical end points. For example, metabolic biomarkers are already measured as part of international regulatory test guidelines, such as triiodothyronine (T3) and thyroxine (T4) hormones as predictors of thyroid toxicity in rodent repeated-dose 90-day studies.^18^ Other metabolic biomarkers, discovered via untargeted metabolomics, include ornithine and cystine for predicting developmental toxicity,^19^ and arachidonic acid, lactic acid, 2′-deoxycytidine and thymidine as predictors of cardiotoxicity.^20^ In ecotoxicology, untargeted metabolomics has discovered metabolites that are predictive of the energetic fitness of marine mussels^21^ and the chronic reproductive toxicity of *Daphnia magna*.^22^ Given these examples of metabolites that are predictive of adversity, and many others described in the MTox700+ biomarker list,^23^ it is important to consider whether metabolic BMDs could be more closely aligned with apical end point BMDs compared to BMDs derived from other molecular assays. Indeed, based on a comparison of NOAELs derived from traditional rodent apical end points versus metabolic responses to 104 chemicals, van Ravenzwaay et al.^24^ reported comparable sensitivity for 75% of cases, increased sensitivity of metabolomics for 8% of the chemicals, and decreased sensitivity for the remaining 17%. Despite BMD modeling being widely applied to transcriptomics data, it has rarely been employed to derive a metabolic BMD. Martinez et al.^14^ applied BMD modeling to metabolomics, transcriptomics and morphological effects in zebrafish eleutheroembryos exposed to tributyltin, revealing similar BMDs for the molecular responses, which were ca. 7-fold lower than the BMDs for morphological traits (based on median BMDL values). Similarly, the distribution of BMDs associated with altered metabolomic features has been shown to be largely comparable with transcriptional BMDs in a HepaRG in vitro liver model exposed to a panel of reference chemicals.^15^

The overall aim of this study was to further explore the application of BMD modeling to in vivo metabolomics data (here meaning polar metabolites and lipids), including to compare the resulting metabolic BMDs to both transcriptional and apical end point BMD values. Through the mechanistic evaluation of the molecular measurements, a further aim emerged to evaluate the capability of metabolomics to simultaneously provide knowledge about the metabolic biotransformations of the exposure chemical. This study builds upon a recent progress by DTT, who have pioneered the application of high throughput transcriptomics and BMD modeling to a 5-day repeated-dose rat assay.^16,17^ The first demonstration of this approach focused on the hepatotoxicant triphenyl phosphate (TPhP),^16^ an organophosphate flame retardant widely used in products such as electronics, automotive equipment and furniture.^25^ Following exposure to five concentrations of TPhP, significant changes in liver weight, clinical chemistry end points and gene expression were observed.^16^ In the study reported here, liver samples from the same TPhP-exposed rats were investigated using ultrahigh-performance liquid chromatography–mass spectrometry (UHPLC–MS) and ^1^H nuclear magnetic resonance (NMR) spectroscopy-based metabolomics. The first objective was to implement a data processing workflow to enable the BMD modeling of the metabolomics data sets to derive metabolic BMD estimates. Next, we sought to compare these findings with the existing transcriptional and apical end point BMDs to evaluate the relative sensitivities of the three datastreams, thereby contributing to the growing body of work investigating the regulatory relevance of ‘omics data. The third objective was to reanalyze the existing transcriptomics data set, four years after its original publication,^16^ leveraging information within the Ingenuity Pathway Analysis knowledgebase (IPA) to generate hypotheses from TPhP-induced changes in upstream gene expression that could be tested using the new metabolomics data set. This led to the final objective, to analyze the metabolomics data set to discover information on the metabolic biotransformations of TPhP using a newly developed workflow,^26^ thereby testing hypotheses generated from the transcriptomics data.

## 2 Methods

### 2.1 Samples from TPhP Exposure Study

Liver tissues from male Sprague–Dawley rats (8–9 weeks of age) were provided by the DTT from a study described previously.^16^ Briefly, the animals were assigned randomly to five dose groups and exposed to TPhP by oral gavage for 4 consecutive days, with corn oil as vehicle: 0, 55, 110, 220, 441, and 881 mg/kg body weight per day of TPhP (hereafter referred to as “mg/kg”). Subsequently, these animals were sacrificed on day 5, and tissues, blood, and serum were collected and flash-frozen. Three liver samples per dose group were obtained for metabolomics analysis (except for the 441 mg/kg dose group, for which only two samples were available; *n* = 17 in the whole study). Liver weight was recorded and a series of clinical chemistry, pathological and transcriptomics assessments were undertaken as reported previously.^16^

### 2.2 Metabolite Extraction from Liver Tissues

Liver tissues (ca. 100 mg each) were prepared using a modified biphasic solvent extraction protocol.^27^ Extraction blanks were prepared by using the same protocol. Briefly, methanol, water (both LC–MS grade, Optima brand, Fisher Scientific) and chloroform (HPLC grade, Fisher Scientific) were prechilled on ice, and then methanol and water (ratio of 2:0.8) were added to each homogenization tube containing frozen liver (16.8 μL/mg wet mass) and homogenized (Precellys-24 bead-based homogenizer, 1.4 mm ceramic beads). Homogenates were transferred to glass vials, and the homogenization tubes were rinsed with further methanol and water, which was also transferred to the vials. Next, chloroform (15 μL/mg) and additional water (7.5 μL/mg) were added to each vial, and the samples were vortexed and placed on wet ice to facilitate the extraction (final solvent ratio 2:2:1.8 methanol/chloroform/water). Subsequently, samples were centrifuged to ensure effective separation of the upper polar layer from the lower lipid layer. Each layer was removed; the polar extracts were split for UHPLC–MS (30% of extract) and NMR (remaining 70%) metabolomics analyses and dried using a centrifugal evaporator (Thermo Savant), and the lipid extracts for UHPLC–MS metabolomics analysis were dried under nitrogen. All extracts were stored at −80 °C until analysis.

### 2.3 ^1^H Nuclear Magnetic Resonance Spectroscopy Data Acquisition and Processing

Polar extracts of the liver samples were resuspended in 650 μL of 150 mM potassium phosphate buffer in D_2_O (pH 7.4) containing 0.58 mM 3-trimethylsilyl-2,2′,3,3′-d4-propionate (TMSP) as a chemical shift reference and 0.2 mM NaN_3_ as an antimicrobial agent. Following transfer to NMR tubes, samples were maintained in an NMR autosampler at 4 °C until analysis. Spectra were measured using an AVANCE IVDr 600 MHz NMR platform and 5 mm room temperature probe (Bruker), applying a standard one-dimensional NOESY presaturation pulse sequence with gradient pulses, using TopSpin 3.5.2 (Bruker) and IconNMR 5.0.2 (Bruker) software. Acquisition parameters included a 9.86 μs 90° pulse, 20 ppm spectral width, 10 ms mixing time, 4-s relaxation delay, and 256 transients collected into 64k data points. Using MetaboLabPy software (version 0.6.4), the NMR spectra were preprocessed by applying an exponential line broadening of 0.3 Hz before Fourier transformation, followed by manual phasing, automated baseline correction, and chemical shift calibrated (TMSP at 0.0 ppm). Next, each spectrum was segmented into 0.005 ppm wide chemical shift bins between 0.2 and 10.0 ppm. Bins representing the residual methanol peak (3.35 to 3.37 ppm) and water peak (4.82 to 4.88 ppm) were removed. The resulting data matrix was then normalized using Probabilistic Quotient Normalization (PQN)^28^ before the application of BMD modeling (Section 2.7). To gain an overview of the metabolic variation within the NMR data set the normalized data was generalized log transformed^29^ prior to visualization using principal component analysis (PCA). NMR peaks were annotated by using the Chenomx NMR software suite (version 8.6, Chenomx, Inc.).

### 2.4 UHPLC–MS Data Acquisition: Metabolomics and Metabolite Identification

Polar extracts of the liver samples were resuspended in acetonitrile/water (75:25) and the lipid extracts in isopropanol/water (75:25); all solvents were LC–MS grade (Optima brand, Fisher Scientific). Intrastudy quality control (QC) samples were prepared by taking an aliquot from each extract, and the pooled QCs (one for polar metabolites and one for lipids) were aliquoted into multiple vials. UHPLC–MS metabolomics analyses were performed using a Dionex UltiMate 3000 Rapid Separation LC system (Thermo Scientific) coupled to a Q Exactive Focus mass spectrometer (Thermo Scientific) with an Accucore 150 Amide HILIC column (100 × 2.1 mm, 2.6 μm, Thermo Scientific) for polar extracts and a Hypersil GOLD C18 column (100 × 2.1 mm, 1.9 μm, Thermo Scientific) for lipid extracts (hereafter termed “LIPIDS”). Mobile-phase composition and flow rate settings were as reported previously.^30^ Data were acquired in both positive and negative electrospray ionization (ESI) modes separately (polar extracts: mass range 70–1050 *m*/*z*; lipid extracts: mass range 150–2000 *m*/*z*) at a resolution of 70,000 (fwhm at *m*/*z* 200), using ExactiveTune 2.8 SP1 (build 2806) software (Thermo Scientific). In total, four UHPLC–MS assays were applied to each sample, hereafter termed “HILIC positive,” “HILIC negative,” “LIPIDS positive,” and “LIPIDS negative.” Columns were conditioned by analyzing 10 intrastudy QC samples at the beginning of the analytical batch. To monitor instrument stability, intrastudy QC samples were also analyzed every fourth LC injection. In addition, extraction blank samples were analyzed to provide data describing the background signals. To annotate and/or identify endogenous metabolites, TPhP and its potential biotransformation products (BTPs), data dependent MS2 in “Discovery mode” was applied to intrastudy QCs over three separate mass ranges for each assay (HILIC: 70–200 *m*/*z*; 200–400 *m*/*z*; 400–1000 *m*/*z*; LIPIDS: 200–400 *m*/*z*; 400–700 *m*/*z*; 700–1500 *m*/*z*), using the following settings: resolution = 17,500; isolation width = 3.0 *m*/*z*; stepped collision energies = 25, 60, 100% (HILIC); or 20, 50, 80% (LIPIDS). To further facilitate the annotation and/or identification of TPhP and its BTPs, additional data dependent MS2 was measured using targeted inclusion lists of putative xenobiotic-related features derived using SyGMa software.^31^ These assays were applied to selected highest-dose liver extracts and preparations of TPhP and diphenyl phosphate (DPhP) analytical standards. Scan ranges were 70–1050 *m*/*z* for HILIC and 200–1500 *m*/*z* for LIPIDS. The following settings were used: MS1 resolution = 35,000; MS2 resolution = 17,500; isolation width 3.0 *m*/*z*; stepped collision energies = 20, 40, 100% (positive ionization); 40, 60, 130% (negative ionization).

### 2.5 Processing UHPLC–MS Endogenous Metabolomics Data and Associated Metabolite Identification

Raw UHPLC–MS data were converted to the mzML format using ProteoWizard^32^ before being deconvoluted using XCMS^33^ to detect individual features. A data matrix of metabolite feature (*m*/*z*-retention time pairs) peak areas was generated for each UHPLC–MS assay.^30^ Features were filtered for quality and retained if they met all of the following criteria: present in >90% of intrastudy QC samples; peak area relative standard deviation < 30% across the QC samples; peak area ratio between the extraction blank and mean intrastudy QC of <5%; and present in >50% of liver extracts. The resulting data matrix was then prepared for statistical analysis using software packages written for the R environment.^34−36^ All matrices were normalized using PQN^28^ to account for small differences in concentration. With the exception of BMD analysis,^37^ univariate analysis including *t*-test and fold-change was applied without further processing. Missing values in the normalized data matrix were imputed using *k*-nearest neighboring features (*k*_NN_, *k* = 5)^38^ before applying BMD analysis. For exploratory visualization of the data using PCA^39^ a generalized logarithmic transform (glog) was applied to the normalized and imputed matrix where the lambda parameter was optimized such that the variance of the QC samples was stabilized across all features. Features were annotated using MS/MS data in conjunction with LipidSearch; Thermo Scientific, Compound Discoverer/mzCloud (Thermo Scientific) and an in-house library of metabolite standards.

### 2.6 Processing UHPLC–MS Xenobiotic Data and Associated Metabolite Identification

After the initial processing of raw UHPLC–MS data using XCMS, as described above, a two-step filter was employed to separate putative xenobiotic-related features from the XCMS output, that is any features arising from TPhP and its BTPs.^26^ First, a “fold-change filter” retained features with ≥10-fold intensity between the median of the highest dose group samples relative to the median of control group samples; next a “dose–response filter” retained only those features exhibiting at least a partial association between the feature intensity and dose level (i.e., linear regression with a normalized slope of between 0.5 and 1.5). This yielded a data matrix (per UHPLC–MS assay) of peak areas for xenobiotic-related features (*m*/*z*-retention time pairs) versus samples. Next the xenobiotic-related features were grouped and putatively annotated, including their ion form and isotopic abundance, using BEAMSpy (5 ppm mass error, 5 s LC retention time tolerance, Pearson correlation coefficient threshold >0.7). Additionally, they were matched against predicted BTPs from SyGMa and, where available, compared against MS/MS measurements of analytical standards (i.e., for TPhP and DPhP). As the liver mass extracted varied across samples, the xenobiotic-related feature intensities were normalized using the same PQN coefficients derived from processing the UHPLC–MS data sets in Section 2.5. This ensured consistency across the processing of the xenobiotic-related and endogenous data sets and maximized the reliability of this step by basing the normalization on thousands of features (not the tens of features associated with TPhP and its BTPs). Missing values in the data matrix were imputed by using the well-established kNN method for dosed biological samples. Since TPhP and its BTPs should not occur in vehicle controls, any missing values in these samples were imputed with a single value equal to half of the lowest intensity feature in the whole study, that is, the lowest intensity across all features from all 4 UHPLC–MS assays, derived in Section 2.5. Finally, the data matrix of many tens of xenobiotic-related features was reduced to a single feature for TPhP and each unique BTP. These representative features were selected based on the following criteria: ensuring experimental MS/MS of the feature was measured; ensuring the LC retention time occurred >30 s (i.e., not in solvent front); maximizing the proportion of MS/MS fragments matching the fragmentation data of TPhP; and maximizing the MS1 peak intensity.

### 2.7 BMD Modeling to Derive Points of Departure

BMD modeling was applied to all five metabolomics data sets: NMR, UHPLC–MS HILIC positive, HILIC negative, LIPIDS positive, and LIPIDS negative. Data were imported into BMDExpress (version 2.3, release-05.01.2021), the data platform was selected as “Generic”, and the BMD analysis type set to ‘EPA BMDS Models (Parametric)’. Two BMD analyses were performed on each metabolomics data set: first, “pre-filter” BMD modeling, followed by BMD modeling to derive points of departure. The *prefilter* BMD modeling used 7 mathematical models [Hill, Power (restrict power was set at ≥1), Linear, Exponential 2, Exponential 3, Exponential 4, and Exponential 5] with BMR set to *3 standard deviations*. While addition of the non-monotonic models were available, we did not feel it was appropriate to employ these models due to the limited number of dose groups in the study, that is, not enough to confidently identify non-monotonic responses. Parameter settings included: maximum iterations = 250; confidence level = 0.95; and an assumption of “constant variance.” Model selection settings included: compute BMDL and BMDU (BMD lower and upper confidence limits, respectively); ignore model nonconvergence; best poly model test based on the model with the lowest Akaike information criterion; *p*-value cutoff of 0.05; flag Hill models that have ‘*k*’ parameters <1/3 of the lowest dose; and exclude the flagged Hill model from the best model selection. The output of this modeling comprised metabolic features that fit a dose–response relationship, and all other metabolic features were rejected. Next, this output was *post-filtered* to retain only those metabolic features whose BMD models met the following criteria: best BMD < highest dose (881 mg/kg); best BMD/BMDL <20 (to remove models with large uncertainty in BMD values, i.e. if BMDL is very low relative to BMD then the model is rejected); and best fit *p*-value > 0.0001 (the higher the *p*-value the better the quality of the fit). The second BMD analysis was conducted only on the subset of metabolic features that had viable dose–response models and passed all three post-filters. The same parameter settings and model selection settings were used, except the BMR was set to *1 standard deviation*. The output was subject to the same set of three post-filters. Next, all five BMD outputs from the 4 UHPLC–MS assays and 1 NMR assay were combined, yielding one list of metabolic features. Finally, consistent with the previous report,^16^ a dose cutoff of 18.3 mg/kg (corresponding to 3 times less than the lowest exposure dose) was applied, and all BMD values below this cutoff were considered below the ‘lower limit of extrapolation’, i.e. are unreliable estimates of points of departure, and reported as <18.3 mg/kg.

### 2.8 Ingenuity Pathway Analysis to Derive Perturbed Pathways and Functions

Analysis of which molecular “canonical pathways” and “toxicological functions” were perturbed following TPhP exposure was conducted using QIAGEN Ingenuity Pathway Analysis (IPA, QIAGEN Inc., https://digitalinsights.qiagen.com/qiagen-ipa). IPA analyses were conducted on liver transcriptomics data (https://cebs.niehs.nih.gov/cebs/data/publication/RR-8), where the gene data had been prefiltered (>1.5-fold-change threshold and significantly changing genes only), and on the liver UHPLC–MS and NMR combined metabolomics data (using the PQN normalized data sets). The metabolomics data were prefiltered to include only significantly changing metabolic features that had been annotated or identified using LipidSearch, Compound Discoverer/mzCloud, and/or an in-house library of metabolite standards. First, data describing the annotated genes and metabolites were imported into IPA, comprising fold-changes (both genes and metabolites) and *p*-values (metabolites only, from *t* tests), comparing each dose to the vehicle control. The transcriptomics data were analyzed using the “core analysis” option named “Tox analysis”; the metabolomics data were analyzed using the “metabolomics analysis” option. The IPA database was used as a reference set and duplicates were identified and removed using the fold-change data. Next, “comparison analysis” was performed for both ‘omics data sets to create (1) canonical pathway and (2) toxicological function heat maps, where these heat maps visualized the activation *z*-scores (of which *z*-scores greater than 2 or less than −2 indicated a significant perturbation from baseline).

### 2.9 Correlation of Xenobiotic Metabolism Gene Expression with Xenobiotic-Related Measurements

Selected correlation analyses of the transcriptomics data set (specifically, genes involved in xenobiotic metabolism and oxidative stress pathways) with the metabolomics data set (specifically, the features discovered to be TPhP and its BTPs) were conducted to explore the finding from the IPA analysis of the gene data that significant xenobiotic metabolism occurred following chemical exposure. Spearman correlation analysis was conducted with significance set at *p*-value < 0.05, and correlations described as “strong” or “weak” based on the absolute value of the correlation coefficient, with 0.6 used as the cutoff.

## 3 Results

### 3.1 BMD Modeling of Metabolomics Data

To characterize the dose-related effects of TPhP in vivo using a metabolomics approach, Sprague–Dawley rats were exposed to five concentrations of this xenobiotic for four consecutive days. Liver tissues were measured for changes in endogenous metabolites and xenobiotic-related compounds using four UHPLC–MS metabolomics assays and an NMR spectroscopy metabolomics assay. Prior to BMD modeling, an initial assessment of the magnitude of metabolic disruption induced by TPhP exposure was performed via principal component analyses of the “endogenous features only” data sets (i.e., the UHPLC–MS data matrix had been filtered to remove all TPhP-related features). All five metabolomics assays revealed clear effects of exposure with the highest dose inducing a particularly large perturbation (Figure S1).

To assess whether metabolic end points (i.e., metabolites) can be useful in determining BMD values for estimating a PoD for human health risk assessment, BMD modeling was applied to all five metabolomics data sets (all features, including endogenous and xenobiotic-related) as described in the Methods section (Section 2.7). This comprised BMD analysis as a prefilter and then repeating the BMD modeling with the BMR set to one standard deviation, together with the application of postfilters. The number of features per assay with “viable” dose–response models is shown in Table 1, revealing a total of 3071 features across all 5 assays. Figure S2 shows which of the mathematical models (Hill, power, linear, exponential 2, exponential 3, exponential 4, and exponential 5) was best for each feature across all the metabolomics assays.

**Table 1:** Number of Features (*m*/*z* Feature in UHPLC–MS Datasets and NMR Bin in NMR Dataset, Including All Endogenous and Xenobiotic-Related Features) Subject to BMD Modeling and Those with Viable Dose–Response Models

assay	total number of endogenous and xenobiotic-related features subject to BMD modeling	total number of features after BMD analysis^a^	total number of endogenous features after BMD analysis^a^	total number of annotated endogenous features after BMD analysis^a^
HILIC positive	3874	635	569	29
HILIC negative	2683	415	377	8
LIPIDS positive	6570	1250	1204	117
LIPIDS negative	3528	680	662	56
NMR^b^	2928	91	91	22
Total	19583	3071	2903	232

a Includes two BMD analyses (BMR of 3SD and then 1SD) and the application of all post-filters.

b Assumes that the analytical sensitivity of NMR was not sufficient to measure TPhP and its biotransformation products.

All TPhP-related features were removed from the UHPLC–MS data sets (leaving in total 2903 endogenous features), and both the UHPLC–MS and NMR endogenous features were annotated, refining the data set further to 232 annotated features (Table 1 and Supporting Information, SII TPhP BMD analysis). Metabolite annotations and/or identifications are essential for interpreting the toxicological effects of TPhP.^40^ The most perturbed annotated metabolites (in terms of fold-change relative to controls), along with the most perturbed clinical chemistry and organ weight end points and genes, are summarized in Table S1.

**Figure 1:**
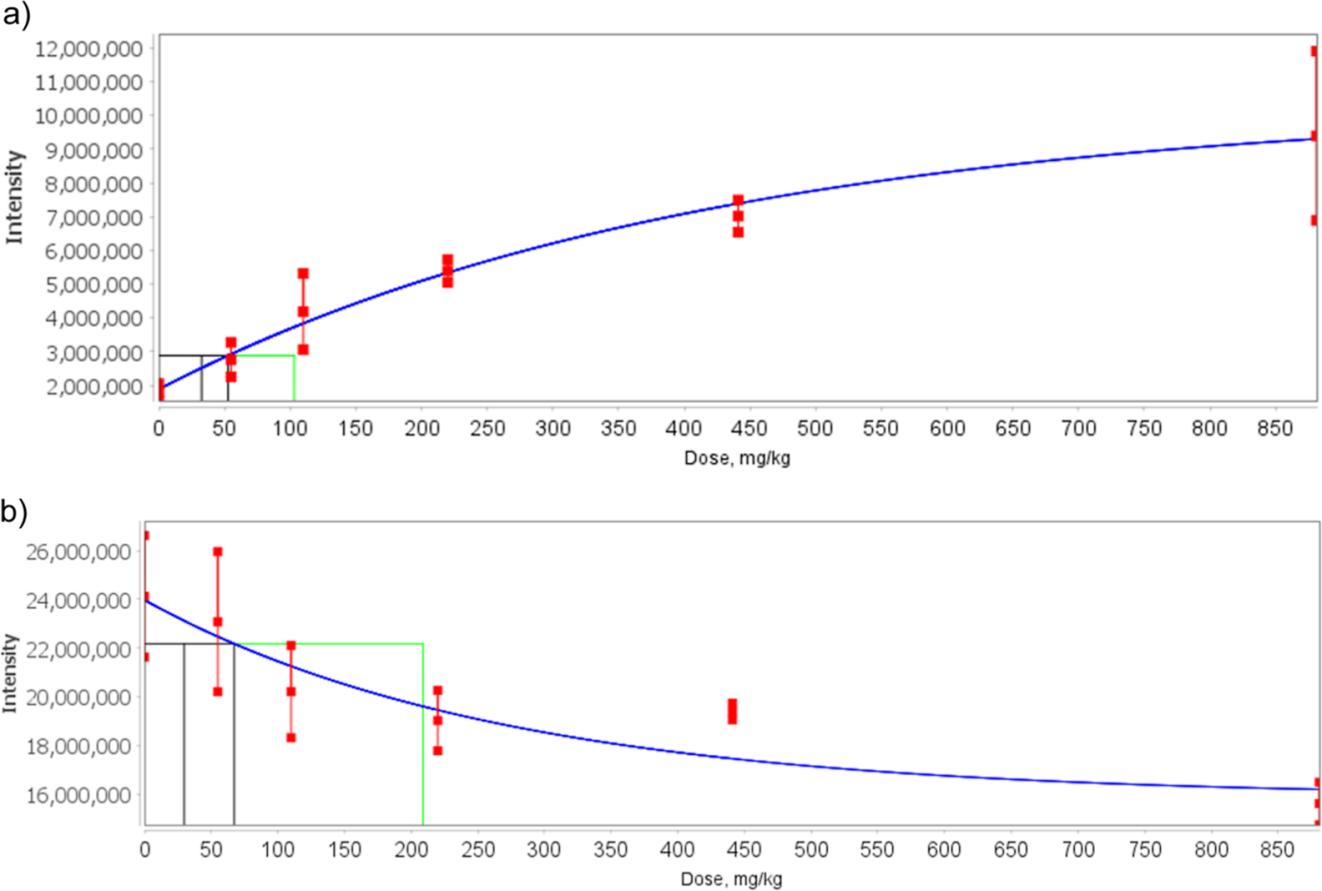
Dose–response curves of putatively annotated endogenous metabolites, (a) UHPLC–MS feature annotated as PE(18:4/18:2); LIPIDS negative assay, and (b) NMR feature annotated as either glucose or maltose; NMR assay. Both features fit the Exponential 4 model. Vertical black lines mark BMDL (BMD lower confidence limit, left) and BMD (right) and a green line shows BMDU (BMD upper confidence limit). Red points indicate mean ± 1 standard deviation for each dose.

Dose–response curves demonstrating BMD, BMDL, and BMDU values for annotated endogenous metabolic features from the BMD modeling are shown in Figure 1. Among the UHPLC–MS annotated endogenous features, the lipid PE(18:4/18:2) (Figure 1a) exhibited the highest fold increase in response to TPhP exposure and has a BMD of 52.7 mg/kg (BMDL of 32.6 mg/kg). In the NMR data set, the feature assigned to glucose or maltose (Figure 1b) showed a significant decrease with a BMD of 67.3 mg/kg (BMDL of 29.8 mg/kg).

**Figure 2:**
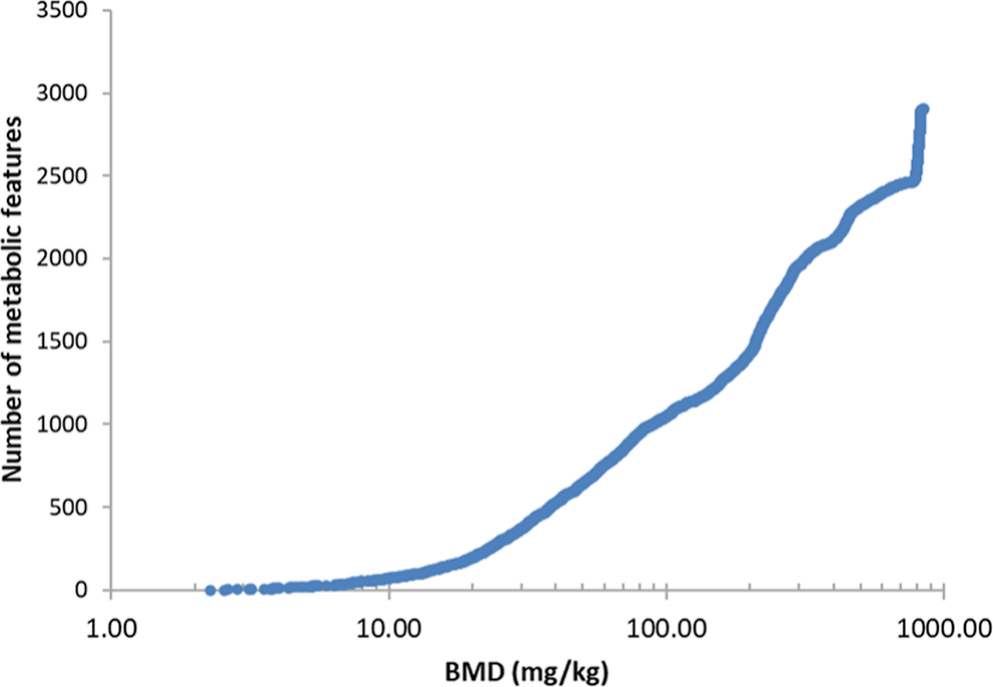
Accumulation plot from the BMD analysis of the rat liver metabolome following TPhP exposure, showing the BMD value for every endogenous metabolic feature that has a viable dose–response model, derived from all five metabolomics assays (four UHPLC–MS and one NMR assay).

An accumulation plot in Figure 2 shows the BMD value for every viable dose–response model plotted against the corresponding endogenous metabolic feature number for that model. The 2903 metabolic features exhibiting dose–response relationships indicate a perturbation to the rat liver metabolome. We cannot discount that an even higher number of metabolic features exhibit dose–response relationships, as our analyses only focused on monotonic models. It is possible that additional metabolite features may fit to nonmonotonic models.

From an examination of the annotated endogenous features with dose–response relationships, it is revealed that lipids account for over 80% of the features with BMD models. The lipid classes altered most significantly (Table S1c) include: LPC (lysophosphatidylcholine), PG (phosphatidylglycerol), SM (sphingomyelin), and TG (triacylglycerol), which mostly showed decreases in intensities with increasing dose, and PC (phosphatidylcholine), PE (phosphatidylethanolamine), PS (phosphatidylserine), and PI (phosphatidylinositol), which exhibited increased intensities with dose level (Figure S3). The majority of perturbed lipid species exhibited sensitive BMD estimates (i.e., departing from baseline at low dose), with TG being an exception. The median BMD (BMDL) values are 47.6 mg/kg (7.0 mg/kg) for PG, 68.9 mg/kg (33.8 mg/kg) for PE, 70.6 mg/kg (35.2 mg/kg) for PC, 81.6 mg/kg (45.1 mg/kg) for PI, 112.6 mg/kg (57.9 mg/kg) for SM, 135.3 mg/kg (53.5 mg/kg) for PS, 160.2 mg/kg (109.0 mg/kg) for LPC, and 818.1 mg/kg (478.9 mg/kg) for TG.

In general, polar metabolic features were less perturbed than lipid species, as reflected by higher (i.e., less sensitive) BMD values. The features participating in galactose metabolism were among the most altered metabolites with BMD (BMDL) values of 67.3 mg/kg (29.8 mg/kg) for glucose (or maltose), 245.1 mg/kg (171.4 mg/kg) for lactose, 589.1 mg/kg (349.4 mg/kg) for galacturonic acid, and 600.1 mg/kg (377.0 mg/kg) for galactonic acid.

### 3.2 Comparison of BMDs from Transcriptomics, Metabolomics, Clinical Chemistry and Apical End Point Data

To determine whether metabolomics analysis provides similar sensitivity end points, the lowest (i.e., most sensitive) BMDs from the current study of the liver metabolome were compared to the previously reported lowest transcriptional BMD (in liver tissues^16^), lowest apical (organ weight) BMD and lowest clinical chemistry BMD (measured in plasma^16^). It is important to note that the transcriptional BMD values are not based on single gene measurements but rather a median of all gene BMDs participating in each Gene Ontology Biological Process (GO BP). There is no equivalent type of ensemble BMDs available for the metabolomics, clinical chemistry, and apical end points; hence, direct comparisons of these data types should be treated with caution.

Table 2 summarizes the lowest BMD values derived from *all* (including unannotated and annotated) metabolic features, *annotated* metabolic features, transcriptional GO BPs, apical (organ weight), and the clinical chemistry end points. These results reveal the sensitivities of each type of ‘omics data set appear to be similar in that they all lie below the lower limit of extrapolation of 18.3 mg/kg, while the clinical chemistry and apical end points are less sensitive. Specifically, the most sensitive clinical chemistry end point - HDL cholesterol, was four times greater than the lower limit of extrapolation (18.3 mg/kg), whereas the most sensitive apical end point—liver weight, was seven times greater than the lower limit of extrapolation. It is notable that many BMD values for both ‘omics approaches are below this lower limit, and as noted previously by the DTT,^16^ additional exposure doses below 55 mg/kg would be required to determine more precise BMD values.

**Table 2:** Comparison of the Most Sensitive (i.e., Lowest Dose) Metabolic, Transcriptional, Clinical Chemistry, and Apical End Point BMDs (mg/kg) Following TPhP Exposure, Including: “All Features” (Unannotated and Annotated) and “Annotated Only” Metabolic BMD Values; Transcriptional Gene Ontology Biological Processes (GO BP) BMDs; and Organ Weight and Clinical End Point BMDs. In the BMD Value Column, the End Point Associated with the BMD Value is Named Where Possible

end point type	BMD value (mg/kg)
(all features) metabolic BMD (liver)	<18.3^a^ (actual lowest BMD value = 2.28 for *m*/*z* = 295.26296)
annotated metabolic BMD (liver)	<18.3 [actual lowest BMD value = 5.16 for LPC (18:2)]
transcriptional GO BP BMD (liver)	<18.3^b^
apical end point BMD (organ weight)	136 (liver weight)
clinical chemistry BMD (plasma)	79 (HDL cholesterol)

a NTP report determined a value cutoff of 18.3 mg/kg, below which all BMD values are deemed below the ‘lower limit of extrapolation’.

b The same value was obtained for 14 GO Biological Processes, see ref (16).

A further contributing factor to the observed differences in sensitivity of the molecular and apical end point BMDs may be related to the exposure duration. The bioassay applied in this study exposed animals to the test item for just 5 days, while repeated dosing studies were of much longer duration (90 days or 2 years), which can explain why the molecular BMDs tend to be significantly more sensitive.

### 3.3 Pathway Analysis of Transcriptomics and Metabolomics Data

The effects of TPhP on canonical pathways and toxicological functions were further examined using IPA, as described in the Methods section (Section 2.8). Both the existing liver transcriptomics^16^ and new liver metabolomics data were analyzed, including only metabolic features annotated to at least Metabolomics Standards Initiative (MSI) level 2.^41^ Of 1365 annotated features in the metabolomics data, 1114 had HMDB or PubChem IDs (others having, for example, LIPID MAPS IDs, which could not be converted to other identifiers), and of these, 848 could be mapped to IPA IDs. After “duplicate removal” by IPA, 294 unique metabolites (of which ∼80% were lipids) remained in the IPA data set. Of the 1436 probes in the transcriptomics data set, only 1172 had gene IDs or gene names, and of these 1160 were mapped to IPA IDs; after duplicate removal, only 1051 genes remained.

**Figure 3:**
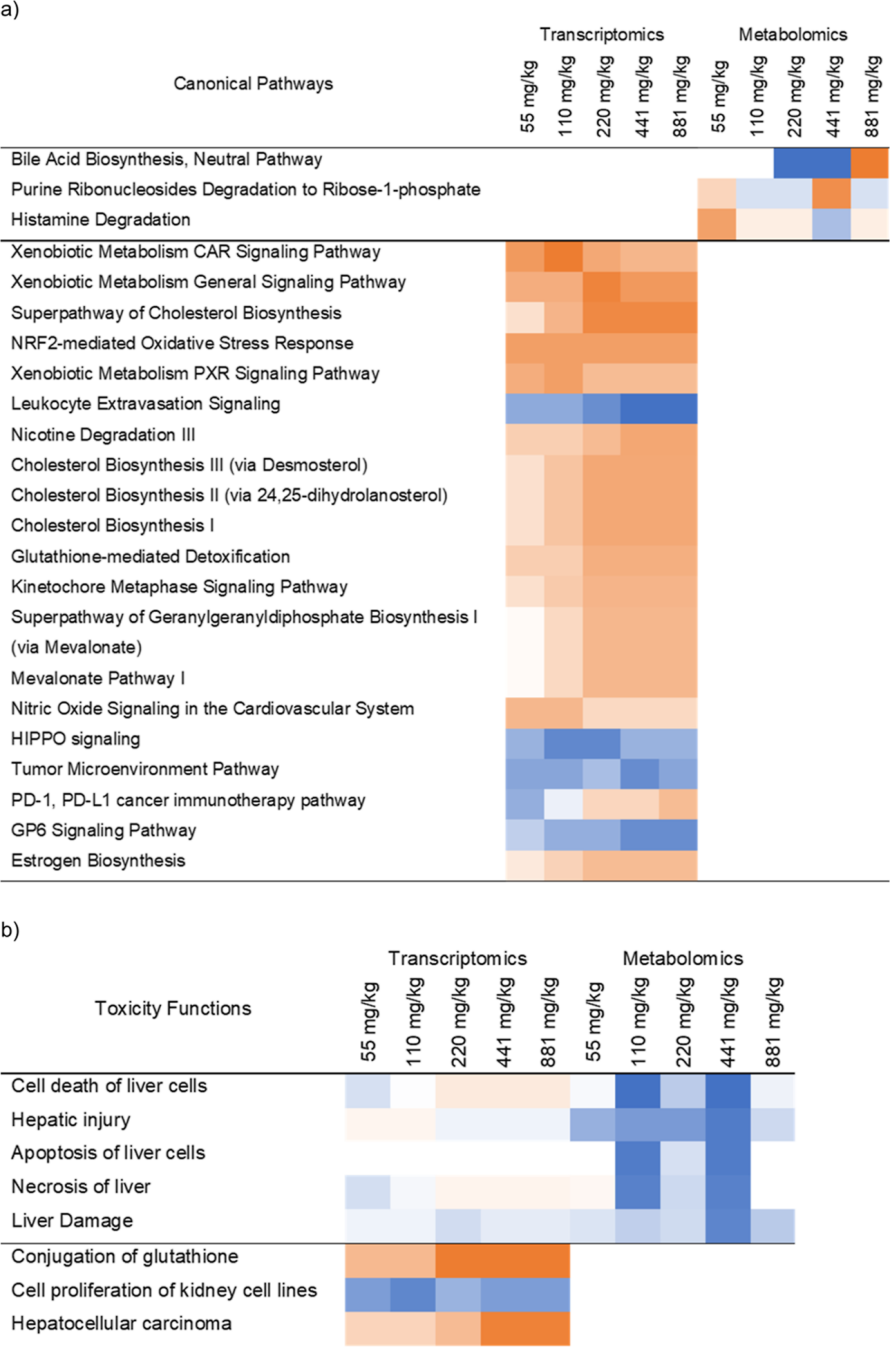
IPA core analysis revealed which (a) canonical pathways and (b) toxicity functions were perturbed following TPhP exposure, utilizing both transcriptomics (left) and metabolomics (right, annotated endogenous features from all five assays) data sets. Orange squares represent activation, and blue squares inhibition, of the pathways and functions as a function of escalating TPhP dose (mg/kg). Only significantly perturbed pathways and functions (with *z*-scores greater than 2 and less than −2 for at least one of the doses) are shown. Pathways and functions are sorted based on maximum absolute *z*-score for each ‘omics approach.

Transcriptomics pathway analysis (Figure 3a) revealed that across the several significantly perturbed pathways, TPhP induced major activation (at all doses) of five pathways associated with xenobiotic metabolism and oxidative stress and four pathways involved in cholesterol biosynthesis. Although the perturbed canonical pathways identified by the metabolomics data generally differed from those identified by the transcriptomics analyses (Figure 3a), the top pathway altered in the metabolomics analysis, bile acid biosynthesis, is closely associated with cholesterol biosynthesis pathways (metabolites and genes from the top 3 pathways for each ‘omics together with their fold-changes are listed in Table S2). In the original study,^16^ cholesterol disruption was identified as one of the major effects of TPhP. An apparent inconsistency between the former and current studies is that, previously, total cholesterol levels were observed to increase with dose, whereas in the new metabolomics data set the annotated cholesterol peaks (Figure S4a) decrease with dose. However, the former clinical chemistry analysis was conducted with plasma, while metabolomics investigated liver tissue. Furthermore, the metabolomics assays specifically measured cholesterol, whereas the clinical chemistry assay detected the total of free cholesterol (∼33%) and cholesterol esters (∼66%).^42^ Similarly, the bile acids identified to MSI level 1 by the metabolomics assays, including glycocholic acid (Figure S4b; decreases with dose) and taurocholic acid (Figure S4c; increases with dose), are each only a component of the total bile salt/acid levels (that were reported to decrease with dose in the original study), again leading to apparent differences that can be explained by considering the specificity of the LC–MS(/MS) metabolomics assays.

Exploring IPA’s toxicity functions (Figure 3b) suggests that TPhP significantly induces liver damage and liver cell death (based on metabolomics data), alters conjugation of glutathione (transcriptomics data), and induces hepatocellular carcinoma (transcriptomics data). Moreover, three glutathione-S-transferase genes involved in the top perturbed transcriptomics pathway—xenobiotic metabolism CAR signaling pathway are the genes that regulate conjugation of glutathione, which in turn plays a major role in oxidative stress response pathways.

### 3.4 Discovery of TPhP Metabolism Tests Hypothesis from IPA Analysis of Upstream Gene Expression

**Figure 4:**
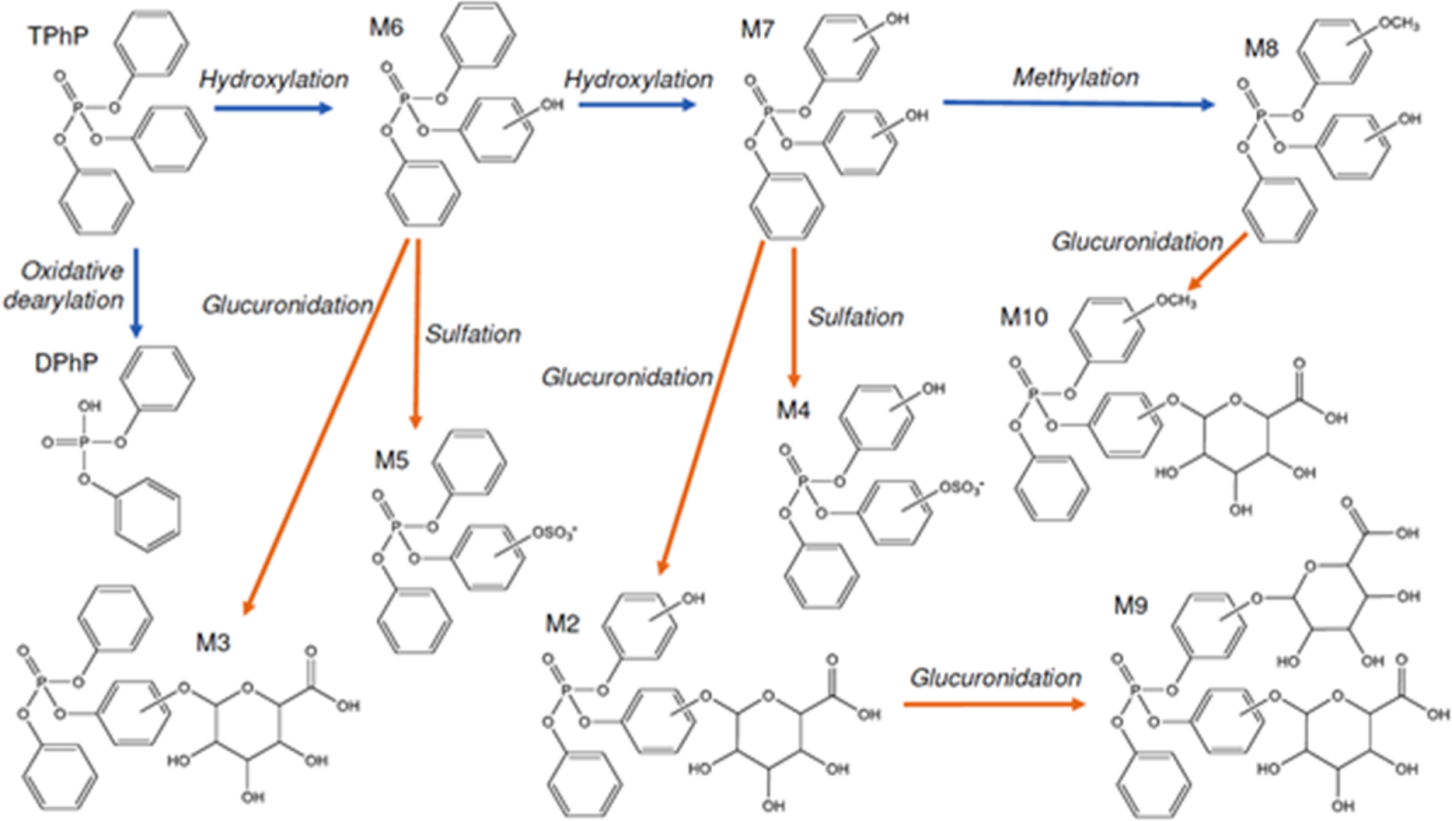
TPhP biotransformation map in rat liver discovered using UHPLC–MS metabolomics assays, with Phase I transformations depicted in blue and Phase II transformations in orange.

An underexploited benefit of metabolomics is its ability to simultaneously measure the endogenous metabolome and xenobiotic-related compounds. This capability of discovering and relatively quantifying xenobiotic biotransformation products was exploited to test some of the hypotheses generated from the IPA core analysis of the transcriptomics data. Applying a data analysis workflow recently developed by Bowen et al.,^26^ 385 features (across all four UHPLC–MS assays) were tentatively annotated as parent TPhP and its BTPs (Table S3). Further analysis of these features, including an examination of extensive MS/MS data, enabled 53 features to be annotated as TPhP and 11 BTPs (Table S4). This list was further reduced (see Section 2.6) to a single representative feature for each of parent TPhP (MSI level 1 identification, confirmed by matching to an analytical standard; Figure S5a,c), its major biotransformation product DPhP (also MSI level 1, confirmed by matching to an analytical standard; Figure S5b,d), and 10 further BTPs (M2-M11), as summarized in Figure 4. This included three phase I BTPs (M6-M8) and six phase II BTPs, while the molecular formula for feature M11 remained unknown.

The relative intensities of these 12 representative features, for TPhP, DPhP, and 10 other BTPs, were investigated to determine which might be associated with the levels of gene expression in the transcriptomics data set. Specifically, Spearman correlation analysis was performed between the TPhP metabolism data and 58 genes participating in xenobiotic metabolism and oxidative stress pathways as derived from the IPA analysis (Table S5).

**Figure 5:**
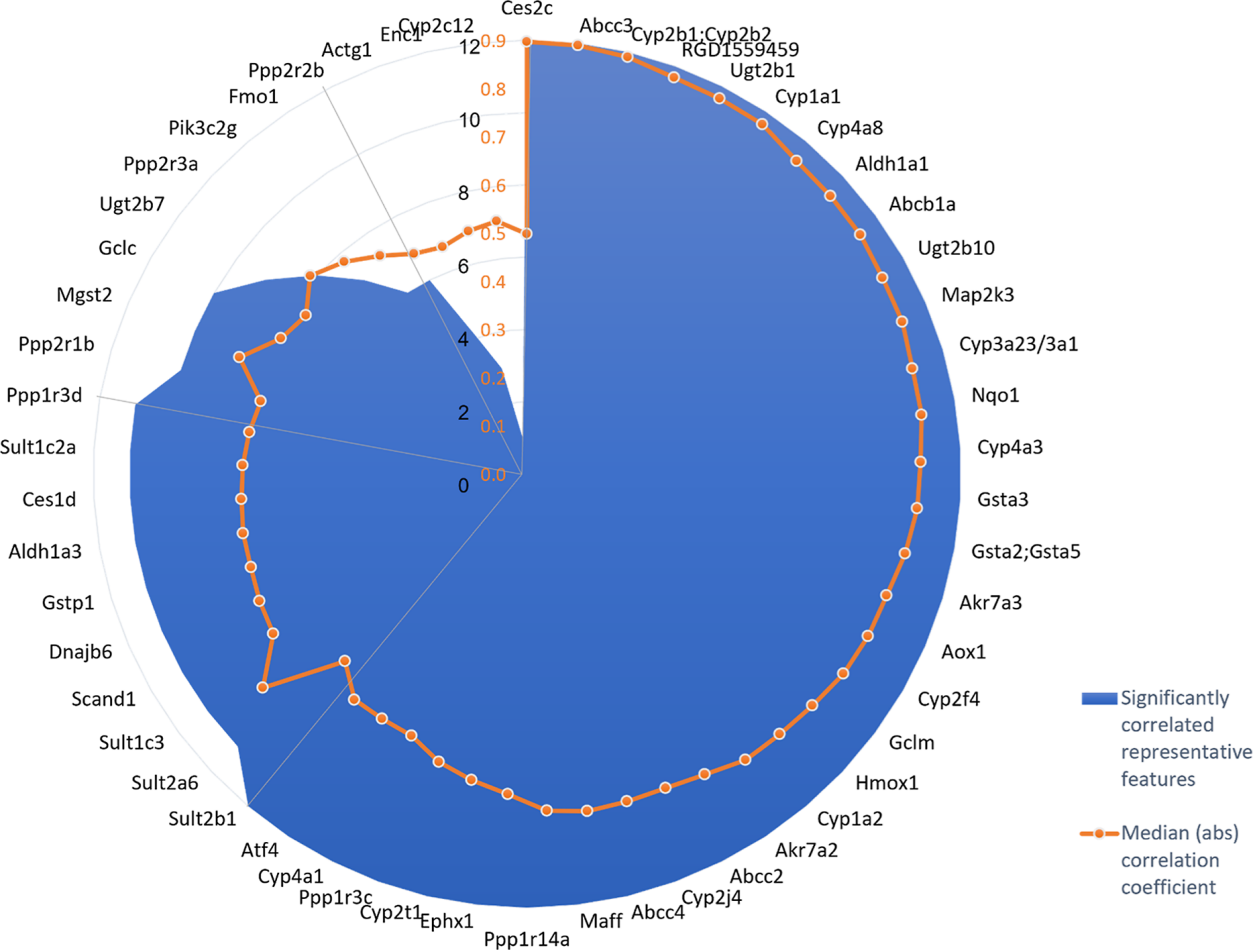
Correlation of 12 representative xenobiotic-related features (measured using UHPLC–MS and corresponding to TPhP, DPhP, and 10 other biotransformation products) with 58 genes participating in xenobiotic metabolism and oxidative stress pathways (xenobiotic metabolism CAR signaling pathway, xenobiotic metabolism general signaling pathway, xenobiotic metabolism PXR signaling pathway and NRF2-mediated oxidative stress response). Filled blue radar plot shows the number of correlated metabolic features for each gene (*p*-value < 0.05). Orange radar plot corresponds to median absolute correlation coefficient (across the 12 features) for the respective genes. Four out of 58 genes exhibited no correlation and are not shown.

The results revealed that xenobiotic metabolism and oxidative stress pathway genes were highly correlated to xenobiotic-related features (Figure 5). Out of 58 xenobiotic metabolism and oxidative stress pathway genes, 59% significantly correlated (*p*-value < 0.05) with all 12 metabolic features (including 10 out of 11 cytochrome P450 (CYP) genes, which represent the largest gene family involved in these pathways) and 64% of the genes exhibited a strong correlation to xenobiotic-related features (above 0.6) based on median absolute correlation coefficient.

## 4 Discussion

Multiple studies have now implemented transcriptional BMD modeling to derive points of departure relevant to setting safety standards for human health.^2,10^ However, to date, few studies have utilized metabolomics data to estimate BMDs.^14,15^ Metabolomics can provide a downstream signature of the biochemical status of a cell or organism, integrating both genetic and environmental factors, and hence could provide BMD values that are similar to traditional apical points of departure. This study applied BMD modeling to UHPLC–MS and NMR data sets to derive BMD estimates for endogenous metabolites after TPhP exposure. It was discovered that 2903 endogenous metabolic features changed in a dose-dependent manner in response to xenobiotic exposure, confirming a considerable impact on the liver metabolome. In a previous toxicometabolomics study of HepaRG liver cells, BMD modeling also revealed a large number of features with viable BMD models, with a higher number of features with BMD estimates observed for hepatotoxicants compared to nonliver injury compounds.^15^ Of the almost 3000 endogenous metabolic features with viable BMD models reported here, 232 could be annotated or identified, limiting the ability to interpret which molecular pathways were perturbed. While metabolite identification has remained a major bottleneck in metabolomics, the recent publication of the MTox700+ metabolic biomarker list could represent a significant step toward a more comprehensive elucidation of chemical modes of action.^23^

An objective of this study was to compare the relative sensitivities of metabolomics-derived BMD estimates to previously published BMDs for transcriptional and apical end points.^16^ Previously, a study of zebrafish eleutheroembryos exposed to tributyltin reported that transcriptomics and metabolomics generate similar BMDs (9.28 and 11.5 nM, respectively), while a morphological BMD exhibited significantly lower sensitivity (67.9 nM).^14^ Similarly, a study in HepaRG cells revealed consistent median BMD values derived from metabolomics and transcriptomics data sets following exposure to chlorpromazine (12.6 and 11.1 μM, respectively), rifampicin (80.1 and 79.7 μM, respectively), ritonavir (12.7 and 13.8 μM, respectively), and tamoxifen (10.9 and 7.3 μM, respectively).^15^ For the case of TPhP, the most sensitive clinical chemistry end point was reported to be HDL cholesterol (BMD of 79 mg/kg), followed by the apical end point—absolute liver weight (BMD of 136 mg/kg).^16^ Using metabolomics, we identified that PG, PE and PC species have some of the most sensitive BMD estimates with median BMD values (considering all species annotated within a given lipid class) of 47.6 mg/kg for PG, 68.9 mg/kg for PE, and 70.6 mg/kg for PC.

Exploring the endogenous metabolome showed that TPhP exposure induced major perturbations to lipids. Multiple lipid classes were shown to be affected including LPC, PG, SM, TG, PC, PE, PS, and PI. These key lipid classes include components of cellular membranes and are known to participate (except SM) in glycerophospholipid/glycerolipid metabolism-regulating processes such as inflammation, immunity and tumor growth.^43^ Van den Eede et al.^44^ previously reported that TPhP causes accumulation of glycerophospholipid and increases palmitoyl-LPC in HepaRG liver cells. Previous studies of flame retardants suggested TGs are elevated in the liver,^45^ however a direct comparison to these results is difficult since only certain TGs (and not the total TG level) were measured in the current study. Despite the polar metabolome being less responsive to TPhP than lipids, perturbations to galactose metabolism (including glucose/maltose, lactose, galacturonic acid, and galactonic acid) were evident. This finding is consistent with an earlier study in zebrafish liver that reported perturbations to carbohydrate metabolism.^46^

Perturbations to cholesterol-associated pathways were evident in both the transcriptomics and metabolomics IPA analysis. One of the top up-regulated genes were carboxylesterase 2C (Ces2c), which is involved in both TG and DG lipase activities,^47^ participates in the hydrolysis or transesterification of xenobiotics, and plays a role in fatty acyl and cholesterol ester metabolism.^48^ Other top upregulated genes included CYP 2b (Cyp2b), which catalyzes xenobiotic metabolism and the synthesis of cholesterol, steroids and other lipids;^49^ and ATP Binding Cassette C3 gene (Abcc3), which plays a role in multidrug resistance and regulates bile salts and bile acids.^50^ While the metabolomics IPA analysis did not identify cholesterol biosynthesis pathways as significantly perturbed, the analysis did reveal that bile acid metabolism is significantly affected, which is a consistent downstream metabolic change with the upstream transcriptional effects on cholesterol metabolism. One challenge in interpreting the TPhP-induced metabolomics responses stems from the fact that ∼80% of the perturbed features arose from lipids, yet the coverage of lipids in canonical pathways and toxicity functions is relatively minimal. Further advances are therefore needed to increase the coverage of lipids in canonical pathways and broaden our knowledge of lipidome perturbations caused by chemicals.

Xenobiotic metabolism and oxidative stress were identified as major effects of TPhP exposure, based on analysis of the transcriptomics data set. Other studies have indicated that TPhP induces oxidative stress, for instance, one multiomics investigation suggested that TPhP induces oxidative stress in human liver cells (L02).^51^ Another study indicated inflammation and apoptosis in mice brain leading to oxidative stress.^52^ It is important to note that IPA is not able to interpret the chemical metabolism data (in part because it does not contain TPhP or its metabolites in the knowledgebase), nor is it able to utilize the metabolomics data to detect perturbations in oxidative stress related pathways (as metabolites do not appear to be associated with oxidative stress pathways in the knowledgebase).

Few studies have previously investigated the metabolism of TPhP. Biotransformation of TPhP in rat liver microsomes was reported to produce only one major BTP–DPhP, via an NADPH-independent mechanism.^53^ A study in human liver microsomes reported the formation of DPhP as well as seven other BTPs, M1-M7, which formed mainly via hydroxylation. The same study also revealed that TPhP is metabolized via phase I cytochrome P450 enzymes, and phase II liver enzymes such as sulfotransferases, glutathione transferases, and glucuronyl transferases.^44^ In the current study, we exploited the ability of metabolomics to discover multiple BTPs of TPhP, and to associate their relative levels with gene expression data, highlighting a novel benefit of measuring metabolomics and transcriptomics data sets from the same liver samples. This revealed that the internal dose of TPhP and its biotransformation products are highly correlated with genes participating in xenobiotic metabolism pathways, with 64% of those genes exhibiting a strong correlation with the xenobiotic and its BTPs. Moreover, strong correlation was established between all 12 xenobiotic-related features and 10 genes from the cytochrome P450 family, including Cyp1a2, which was previously shown to be a key CYP in the metabolism of TPhP.^54^ However, the previous study did not investigate the link between TPhP and Cyp2b or Cyp1a1 or Cyp4a8, for which in this study the correlation was substantially stronger. These results demonstrate that xenobiotic-related features simultaneously measured with endogenous metabolites can provide valuable insights into the toxicity mechanism.

Collectively, these findings add more evidence that BMD modeling can be successfully applied to metabolomics data, here revealing a unique pattern of lipid and polar metabolite disruptions in the liver after exposure to TPhP. It was demonstrated that metabolomics-derived BMD estimates are similarly sensitive to those obtained from modeling transcriptional responses, and more sensitive than both clinical chemistry and apical end point BMD values. Analysis of both the metabolomics and transcriptomics data sets revealed that a perturbation to cholesterol (and downstream) pathways was one of the major effects of TPhP exposure, consistent with clinical chemistry measurements. An investigation of xenobiotic-related features in the metabolomics data set led to the discovery of 11 biotransformation products of TPhP, and also showed that the levels of TPhP and its BTPs are highly correlated with multiple genes participating in xenobiotic metabolism pathways. These findings could contribute toward testing in OECD Test Guideline 417 (toxicokinetics),^55^ providing some information on the presence of the test substance and its biotransformation products. Ultimately, this study adds to the growing body of publications demonstrating the value of metabolomics and transcriptomics for estimating mechanistically anchored points of departure that are relevant to human risk assessment.

## Supporting Information Available

Supporting endogenous and xenobiotic metabolomics results, TPhP BMD analysis results (PDF)
(XLSX)
