# Supplementary material for: Metabolomics Simultaneously Derives Benchmark Dose Estimates and Discovers Metabolic Biotransformations in a Rat Bioassay
Source: Chem Res Toxicol. 2024 Jun 6;37(6):923–34. doi: 10.1021/acs.chemrestox.4c00002 (PMC11187623; doi:10.1021/acs.chemrestox.4c00002)
Supplement: Supplementary file 1 — tx4c00002_si_001.pdf [file tx4c00002_si_001.pdf]

Supplementary information SI: Supporting endogenous and xenobiotic metabolomics results, including tables and figures for the manuscript - Metabolomics simultaneously derives benchmark dose estimates and discovers metabolic biotransformations in a rat bioassay

Elena Sostare[1], Tara J. Bowen[2], Thomas N. Lawson[1], Anne Freier[2], Xiaojing Li[2], Gavin R. Lloyd[3], Lukáš Najdekr[3**], Andris Jankevics[3], Thomas Smith[3], Dorsa Varshavi[3], Christian Ludwig[3], John K. Colbourne[1,2], Ralf J. M. Weber[1,2,3], David M. Crizer[4], Scott S. Auerbach[4], John R. Bucher[4] and Mark R. Viant[1,2,3*]

[1] Michabo Health Science Ltd., Union House, 111 New Union Street, Coventry, CV1 2NT, United Kingdom

[2] School of Biosciences, University of Birmingham, Birmingham, B15 2TT, United Kingdom

[3] Phenome Centre Birmingham, University of Birmingham, Birmingham, B15 2TT, United Kingdom

[4] Division of Translational Toxicology, National Institute of Environmental Health Sciences, Research Triangle Park, North Carolina, USA

* Corresponding author (Mark R. Viant)

E-mail: mark@michabo.co.uk

Phone: +44 0121 414 2219

Address: Michabo Health Science Ltd., Union House, 111 New Union Street, Coventry, CV1 2NT, United Kingdom

**Current address: Institute of Molecular and Translational Medicine, Palacký University Olomouc, Hněvotínská 5, 77900 Olomouc, Czech Republic

TABLE OF CONTENTS

Figure S1. Principal component analysis scores plots showing the effect of TPhP-exposure on the rat liver metabolome. S3

Figure S2. Best model counts for all features across the four UHPLC-MS (HILIC positive, HILIC negative, LIPIDS positive and LIPIDS negative) and one NMR datasets. S5

Table S1. Most perturbed - in terms of fold-change relative to controls - (a) clinical chemistry and organ weight endpoints, (b) differentially expressed genes (up in red, down in green), and (c) annotated metabolic features, sorted by BMD values. S6

Figure S3. Relative intensities (%) of lipid species measured in rat liver at each TPhP exposure dose. S10

Table S2. Metabolite and gene fold-changes in the top 3 canonical pathways revealed by IPA analysis of the liver transcriptome and metabolome data following exposure to TPhP. S11

Figure S4. Spectral matching for selected metabolic features - a) cholesterol, b) glycocholic acid, c) taurocholic acid measured in the rat liver metabolome (“query”) and also as analytical standards (“library”), allowing Metabolomics Standards Initiative level 1 identifications. S13

Table S3. Number of features measured in the 4 UHPLC-MS assays that were annotated - with varying levels of confidence - as parent TPhP or its biotransformation products, across different stages of data processing. S14

Table S4. Summary of UHPLC-MS metabolomics measurements of TPhP and its biotransformation products, measured in rat liver. S15

Figure S5. Confirmation of MSI level 1 identification of (a and c) TPhP and (b and d) its primary biotransformation product DPhP by comparison to analytical standards. S21

Table S5. Summary of Spearman correlation analysis of the relative intensities of 12 representative features (for TPhP, DPhP and 10 other BTPs) with the levels of gene expression reported by the NTP. S22

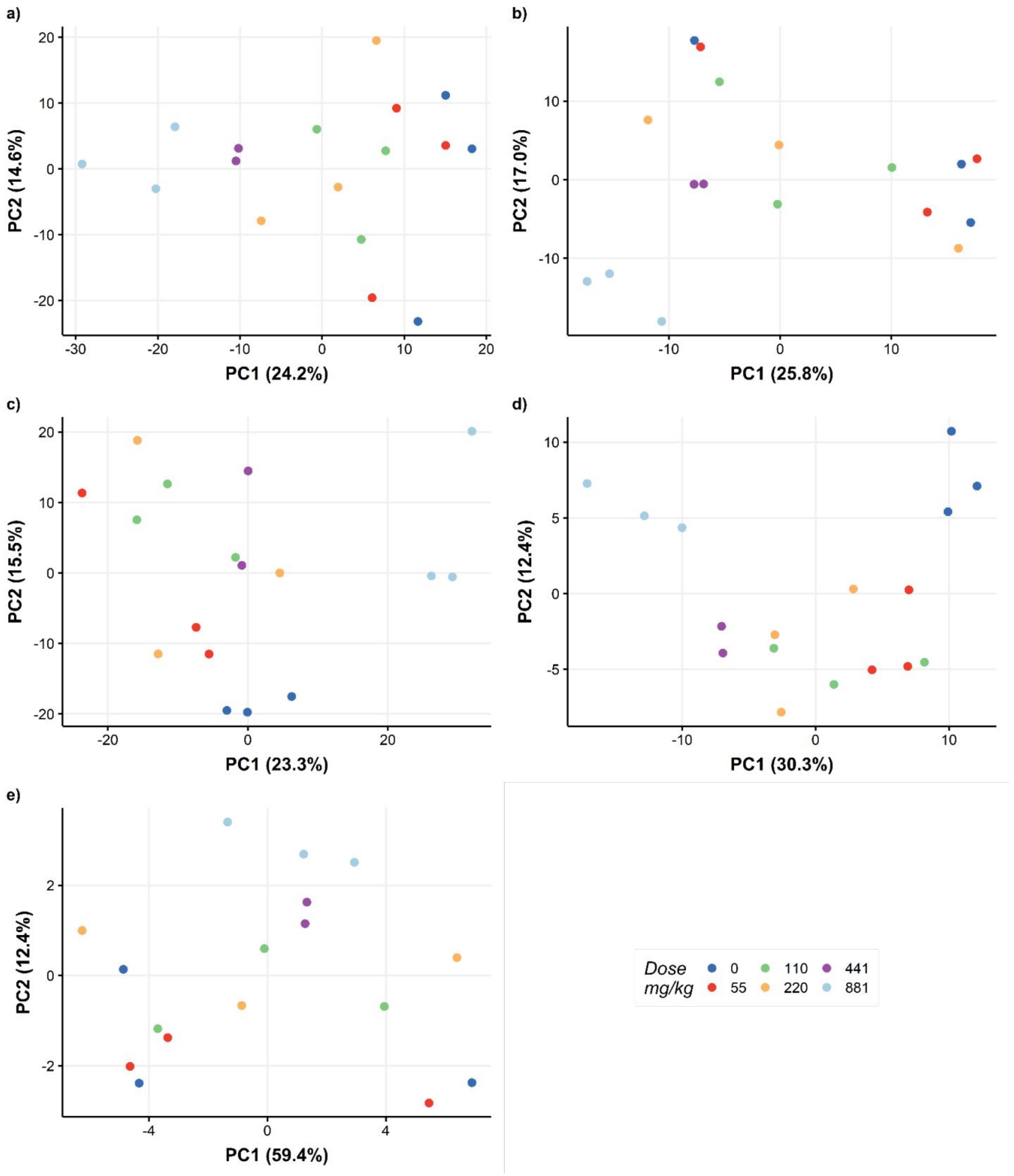
a)
PC1 (24.2%)
PC2 (14.6%)
b)
PC1 (25.8%)
PC2 (17.0%)
c)
PC1 (23.3%)
PC2 (15.5%)
d)
PC1 (30.3%)
PC2 (12.4%)
e)
PC1 (59.4%)
PC2 (12.4%)
Dose mg/kg
0
55
110
220
441
881

Figure S1. Principal component analysis scores plots showing the effect of TPhP-exposure on the rat liver metabolome, measured using multiple UHPLC-MS and NMR spectroscopy based metabolomic assays: (a) UHPLC-MS HILIC positive, (b) UHPLC-MS HILIC negative, (c) UHPLC-MS LIPIDS positive, (d) UHPLC-MS LIPIDS negative, and (e) ^{1}H NMR datasets. The control is indicated with dark blue dots and all other colours correspond to the TPhP dose (mg/kg).

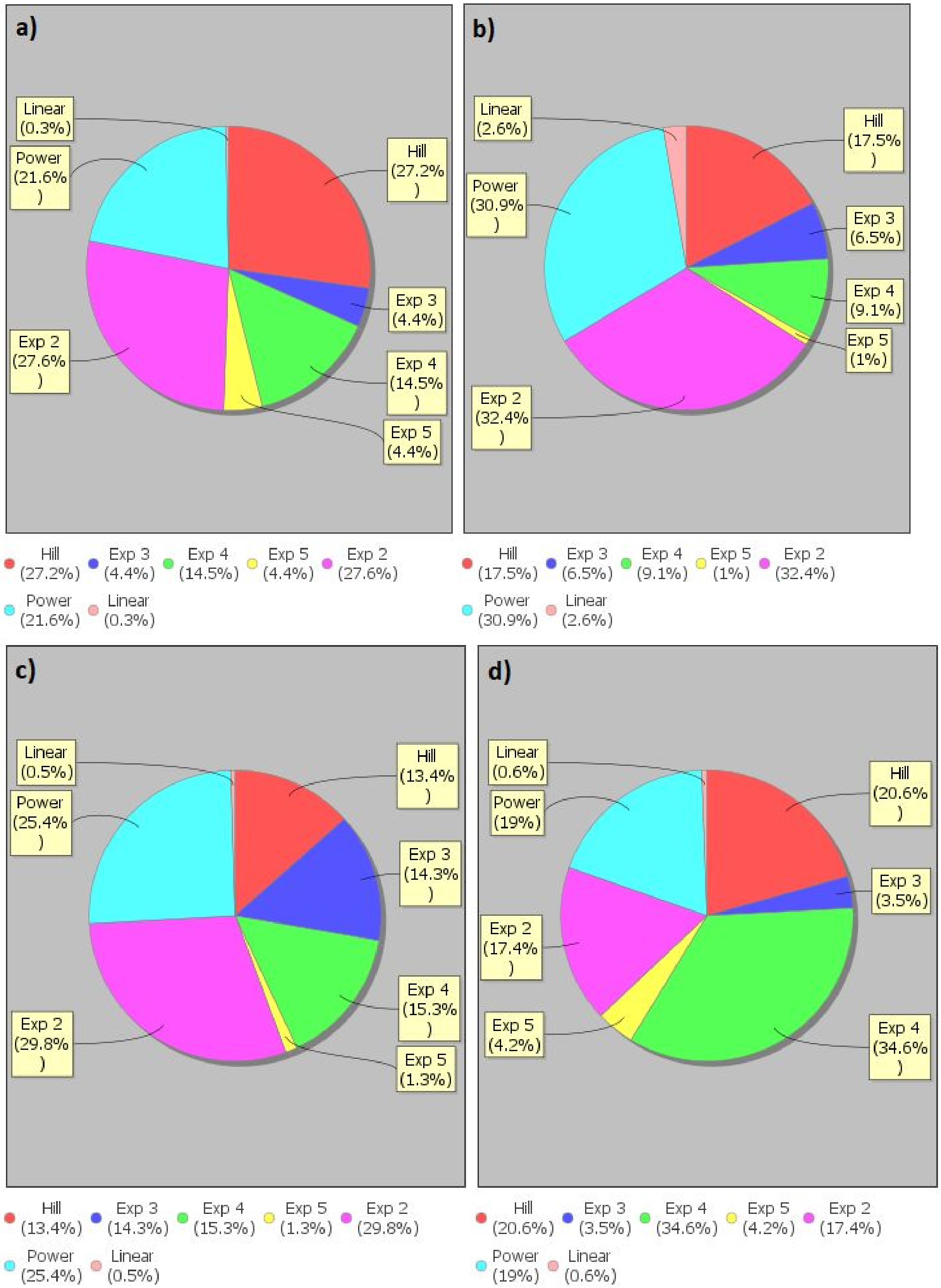
a)
Linear (0.3%)
Power (21.6%)
Hill (27.2%)
Exp 3 (4.4%)
Exp 2 (27.6%)
Exp 4 (14.5%)
Exp 5 (4.4%)
Hill (27.2%) Exp 3 (4.4%) Exp 4 (14.5%) Exp 5 (4.4%) Exp 2 (27.6%) Power (21.6%) Linear (0.3%)
b)
Linear (2.6%)
Hill (17.5%)
Power (30.9%)
Exp 3 (6.5%)
Exp 4 (9.1%)
Exp 5 (1%)
Exp 2 (32.4%)
Hill (17.5%) Exp 3 (6.5%) Exp 4 (9.1%) Exp 5 (1%) Exp 2 (32.4%) Power (30.9%) Linear (2.6%)
c)
Linear (0.5%)
Hill (13.4%)
Power (25.4%)
Exp 3 (14.3%)
Exp 4 (15.3%)
Exp 2 (29.8%)
Exp 5 (1.3%)
Hill (13.4%) Exp 3 (14.3%) Exp 4 (15.3%) Exp 5 (1.3%) Exp 2 (29.8%) Power (25.4%) Linear (0.5%)
d)
Linear (0.6%)
Power (19%)
Hill (20.6%)
Exp 3 (3.5%)
Exp 2 (17.4%)
Exp 5 (4.2%)
Exp 4 (34.6%)
Hill (20.6%) Exp 3 (3.5%) Exp 4 (34.6%) Exp 5 (4.2%) Exp 2 (17.4%) Power (19%) Linear (0.6%)

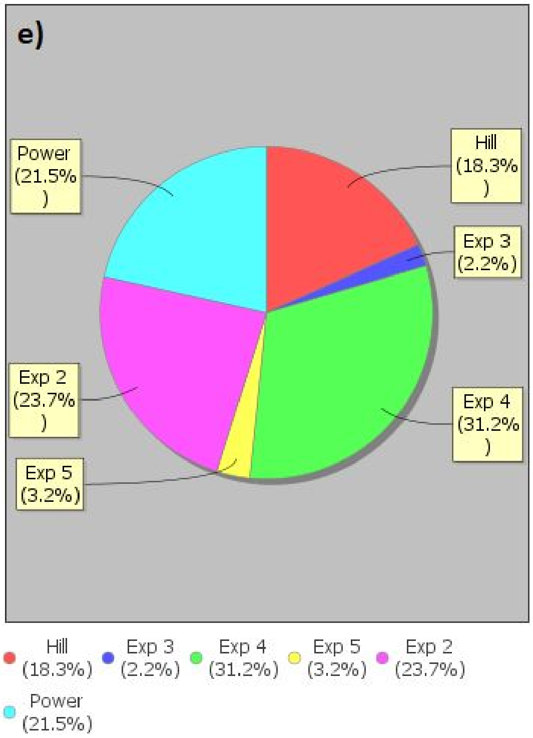

Figure S2. Best model counts for all features across the four UHPLC-MS (HILIC positive, HILIC negative, LIPIDS positive and LIPIDS negative) and one NMR datasets.

*Table S1. Most perturbed - in terms of fold-change relative to controls - (a) clinical chemistry and organ weight endpoints, (b) differentially expressed genes (up in red, down in green), and (c) annotated metabolic features, sorted by BMD values. Clinical chemistry and transcriptomics data were adapted from a previous study [16]. Negative sign in fold changes indicates a decrease relative to the control group. *Previous study [16] determined a value cutoff of 18.3 mg/kg (3 times below the lowest exposure dose), below which all BMD values are deemed below the lower limit of extrapolation; **Significant fold-changes (t-test p-value <0.05) in metabolomics data are reported in bold.*

a)

			Fold-changes				
Clinical chemistry and organ weight endpoints	BMD (mg/kg)	BMDL (mg/kg)	55 mg/kg	110 mg/kg	220 mg/kg	441 mg/kg	881 mg/kg
HDL Cholesterol	79	39	0.25	0.30	0.40	0.62	0.77
Liver Weight Relative	103	71	0.07	0.07	0.16	0.24	0.45
Liver Weight Absolute	136	48	0.05	0.05	0.18	0.17	0.19
Cholesterol	142	90	0.19	0.28	0.34	0.53	0.75
A/G Ratio	147	103	-0.08	-0.04	-0.11	-0.18	-0.25
Free Thyroxine	178	139	0.00	-0.13	-0.26	-0.33	-1.45
LDL Cholesterol	213	123	0.00	0.09	0.13	0.10	0.59
Globulin	328	174	0.03	0.01	0.09	0.14	0.12
Terminal Body Weight (SD4)	486	278	-0.03	-0.02	0.01	-0.07	-0.25
Albumin	576	322	-0.03	-0.03	-0.02	-0.03	-0.14

b)

				Fold-changes				
Gene	Affymetrix	**BMD**	(BMDL – BMDU)	55 mg/kg	110 mg/kg	220 mg/kg	441 mg/kg	881 mg/kg
Ces2c;LOC100910040	1368905_at	<18.3*	-	2.3	2.8	4.4	10.3	16.3
Cyp2b1; Cyp2b2; LOC100909962	1371076_at	<18.3*	-	3.1	5.5	6.9	9	10.5
–	1397924_at	<18.3*	-	2.2	2.9	3.4	6.7	5.8
Abcc3	1369698_at	48	(26–80)	1.3	2.8	5.6	9.8	12.7

Per2	1368303_at	48	(36–73)	2.1	2.8	3.3	7.2	4.6
–	1381811_at	58	(42–94)	1.2	2.2	2.6	5.9	2.1
Gsta3	1371089_at	84	(57–152)	1.1	1.4	2.5	4.6	6.6
Cyp1a1	1370269_at	153	(115–278)	1.2	1.2	1.6	3	6.1
Akr1b7	1368569_at	238	(186–329)	-1.4	1.1	2.5	2.9	29
Aldh1a7	1368718_at	414	(263–962)	-1.8	4.2	8.6	9.5	69.4
Scd; Scd1	1370355_at	<18.3*	-	-3.8	-6.9	-6	-10.5	-11.1
Aoc3; G6pc; Psme3	1370725_a_at	27	(16–53)	-2	-2.3	-3.8	-5.1	-4.5
Rprm	1390672_at	59	(42–95)	-2.2	-2.2	-2.9	-5.9	-3.3
Stac3	1395403_at	81	(56–143)	-1.4	-1.5	-2.4	-7	-11.6
Dhrs7; LOC10036439	1397205_at	163	(118–255)	1.9	1.2	-1.3	-2.4	-7.7
Car3	1386977_at	201	(141–332)	-1	-1.1	-1.6	-2.5	-9.8
Lox	1368171_at	208	(122–262)	-1.1	1.3	-2.7	-5.4	-5.2
Lox	1368172_a_at	210	(119–306)	-1.4	1.1	-2.7	-4.7	-5.1
Serpina7	1371143_at	330	(167–546)	-1.1	-1.3	-1.4	-1.5	-5.1
LOC100134871; LOC689064	1371102_x_at	485	(273–825)	-1.2	-1	-1.1	-1.4	-5.4

c)

					Fold-changes**				
Name	Probe ID	Assay	BMD	(BMDL-BMDU)	55 mg/kg	110 mg/kg	220 mg/kg	441 mg/kg	881 mg/kg
PG(18:0/20:4)	M798T481	LIPIDS negative	<18.3	-	2.1	**2.1**	**2.9**	**2.8**	**3.2**
PE(14:0/22:6)	M736T440	LIPIDS positive	47.0	(29.1-91.9)	1.6	2.4	**3.1**	**4.0**	**5.1**
PE(18:4/18:2)	M734T440	LIPIDS negative	52.7	(32.6-103.1)	1.5	2.3	**3.0**	**3.9**	**5.2**
PE(14:0/20:4)	M712T450	LIPIDS positive	54.6	(32.5-113.1)	1.2	1.8	**2.1**	2.7	**3.3**
PS(40:6)	M837T301	HILIC positive	70.5	(20.2-309.6)	2.1	2.6	2.7	**4.4**	4.4

PI(18:0/18:2)	M862T303	HILIC negative	81.6	(39.5-230.4)	1.3	1.7	1.9	**2.9**	2.9
PI(36:2)	M881T303	HILIC positive	94.0	(49.2-236.5)	1.4	1.6	1.8	**3.1**	3.3
PE(14:0/20:4)	M710T450	LIPIDS negative	155.6	(114.9-272.2)	1.3	1.6	**1.9**	2.6	**3.4**
PC(30:2)	M703T426	LIPIDS positive	161.3	(118.7-355.7)	1.1	1.4	1.3	2.2	**2.9**
Galactonic acid	M391T498	HILIC negative	600.1	(377.0-802.9)	**-2.8**	**-2.2**	-1.7	-1.4	**4.1**
SM(t42:0)	M878T558	LIPIDS negative	35.0	(23.8-59.4)	-1.0	**-1.2**	**-1.4**	**-1.9**	**-2.3**
SM(t42:0)	M834T557	LIPIDS positive	108.6	(67.2-167.7)	1.1	-1.0	**-1.2**	**-1.7**	**-2.2**
PC(19:0/22:6)	M893T508	LIPIDS negative	151.5	(112.1-344.7)	-1.1	-1.3	-1.2	**-1.6**	**-2.9**
PC(41:6)	M849T507	LIPIDS positive	185.3	(134.5-442.0)	-1.2	-1.3	-1.2	**-1.5**	**-2.6**
PC(40:4)	M839T528	LIPIDS positive	186.5	(113.3-388.7)	-1.3	-1.1	**-1.7**	**-1.4**	**-2.5**
PG(46:2)	M916T579	LIPIDS positive	190.5	(121.7-370.1)	**-1.4**	**-1.5**	**-1.4**	-1.4	**-2.3**
LPC(22:6)	M590T104	LIPIDS positive	196.2	(118.7-414.4)	-1.2	-1.3	-1.3	-1.6	**-2.4**
PC(22:4/20:4)	M903T476	LIPIDS negative	212.1	(133.1-428.4)	**-1.3**	-1.2	-1.2	-1.3	**-2.3**
PC(42:7)	M861T497	LIPIDS positive	237.0	(146.7-497.4)	-1.3	-1.2	-1.1	-1.3	**-2.1**
TG(26:0/18:2/18:2)	M1013T656	LIPIDS positive	814.2	(495.7-835.5)	-2.0	-2.3	-2.0	-2.5	2.0

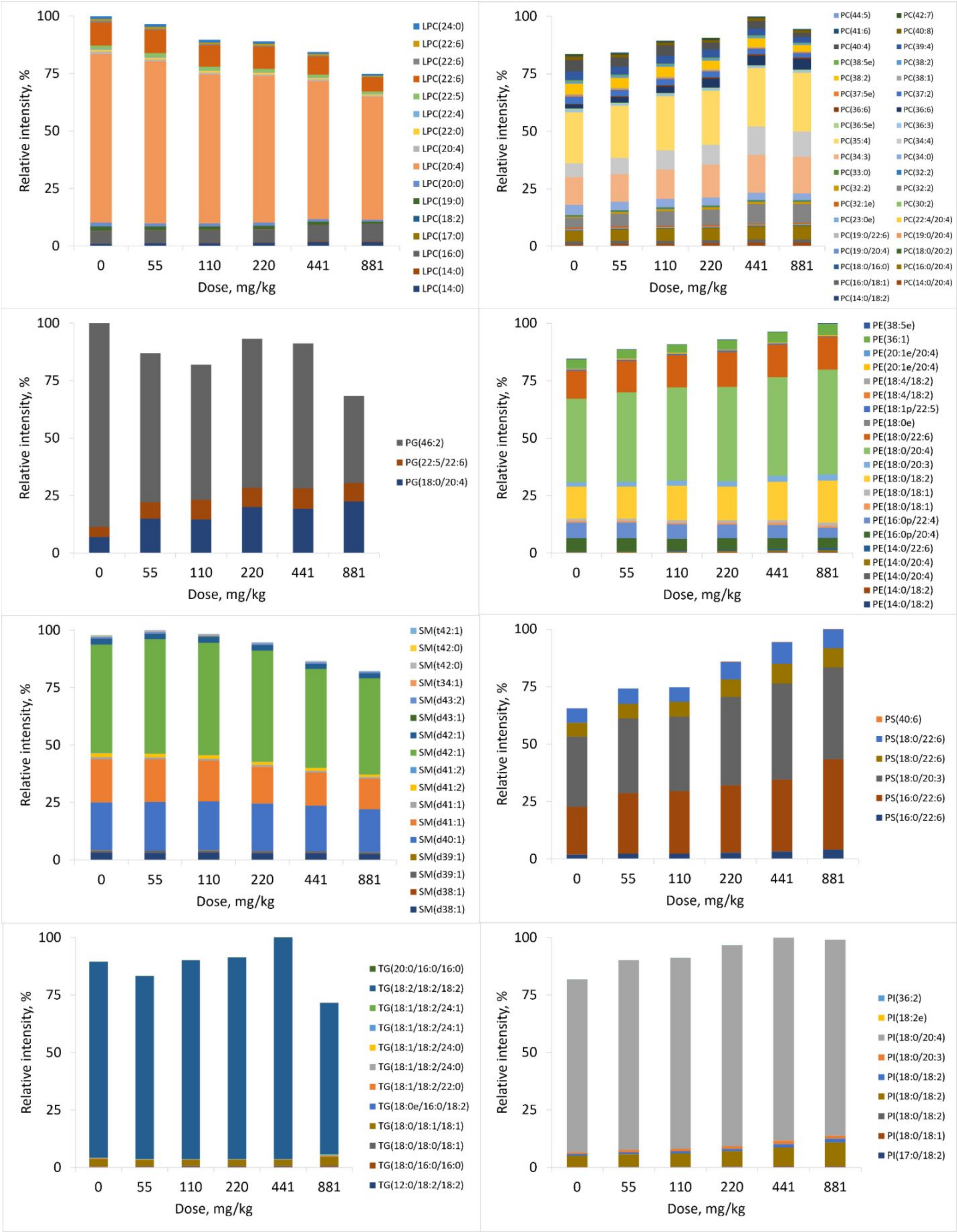

Relative intensity, %
Dose, mg/kg
0
55
110
220
441
881
100
75
50
25
LPC(24:0)
LPC(22:6)
LPC(22:6)
LPC(22:6)
LPC(22:5)
LPC(22:4)
LPC(22:0)
LPC(20:4)
LPC(20:4)
LPC(20:0)
LPC(19:0)
LPC(18:2)
LPC(17:0)
LPC(16:0)
LPC(14:0)
LPC(14:0)
PC(44:5)
PC(42:7)
PC(41:6)
PC(40:8)
PC(40:4)
PC(39:4)
PC(38:5e)
PC(38:2)
PC(38:2)
PC(38:1)
PC(37:5e)
PC(37:2)
PC(36:6)
PC(36:6)
PC(36:5e)
PC(36:3)
PC(35:4)
PC(34:4)
PC(34:3)
PC(34:0)
PC(33:0)
PC(32:2)
PC(32:2)
PC(32:2)
PC(32:1e)
PC(30:2)
PC(23:0e)
PC(22:4/20:4)
PC(19:0/22:6)
PC(19:0/20:4)
PC(19:0/20:4)
PC(18:0/20:2)
PC(18:0/16:0)
PC(16:0/20:4)
PC(16:0/18:1)
PC(14:0/20:4)
PC(14:0/18:2)
PG(46:2)
PG(22:5/22:6)
PG(18:0/20:4)
PE(38:5e)
PE(36:1)
PE(20:1e/20:4)
PE(20:1e/20:4)
PE(18:4/18:2)
PE(18:4/18:2)
PE(18:1p/22:5)
PE(18:0e)
PE(18:0/22:6)
PE(18:0/20:4)
PE(18:0/20:3)
PE(18:0/18:2)
PE(18:0/18:1)
PE(18:0/18:1)
PE(16:0p/22:4)
PE(16:0p/20:4)
PE(14:0/22:6)
PE(14:0/20:4)
PE(14:0/20:4)
PE(14:0/18:2)
PE(14:0/18:2)
SM(t42:1)
SM(t42:0)
SM(t42:0)
SM(t34:1)
SM(d43:2)
SM(d43:1)
SM(d42:1)
SM(d42:1)
SM(d41:2)
SM(d41:2)
SM(d41:1)
SM(d41:1)
SM(d40:1)
SM(d39:1)
SM(d39:1)
SM(d38:1)
SM(d38:1)
PS(40:6)
PS(18:0/22:6)
PS(18:0/22:6)
PS(18:0/20:3)
PS(16:0/22:6)
PS(16:0/22:6)
TG(20:0/16:0/16:0)
TG(18:2/18:2/18:2)
TG(18:1/18:2/24:1)
TG(18:1/18:2/24:1)
TG(18:1/18:2/24:0)
TG(18:1/18:2/24:0)
TG(18:1/18:2/22:0)
TG(18:0e/16:0/18:2)
TG(18:0/18:1/18:1)
TG(18:0/18:0/18:1)
TG(18:0/16:0/16:0)
TG(12:0/18:2/18:2)
PI(36:2)
PI(18:2e)
PI(18:0/20:4)
PI(18:0/20:3)
PI(18:0/18:2)
PI(18:0/18:2)
PI(18:0/18:2)
PI(18:0/18:1)
PI(17:0/18:2)

Figure S3. Relative intensities (%) of lipid species measured in rat liver at each TPhP exposure dose. Each bar represents a percentage of the sum of averaged lipid intensities (derived from PQN normalised datasets) for the following lipid classes: LPC (lysophosphatidylcholine), PG (phosphatidylglycerol), SM (sphingomyelin), TG (triacylglycerol), PC (phosphatidylcholine), PE (phosphatidylethanolamine), PS (phosphatidylserine) and PI (phosphatidylinositol). Relative intensity was normalised based on the highest bar in each chart (representing 100% for a particular lipid class).

Table S2. Metabolite and gene fold-changes in the top 3 canonical pathways revealed by IPA analysis of the liver transcriptome and metabolome data following exposure to TPhP. N/A - not applicable for the type of analysis (e.g., gene measurements are not applicable for metabolomics).

			Transcriptomics					Metabolomics				
Rank	Canonical Pathways	Molecular entity	55 mg/kg	110 mg/kg	220 mg/kg	441 mg/kg	881 mg/kg	55 mg/kg	110 mg/kg	220 mg/kg	441 mg/kg	881 mg/kg
M1	Bile Acid Biosynthesis, Neutral Pathway	**AMP**	N/A	N/A	N/A	N/A	N/A	1.34	1.54	-1.14	1.39	1.34
M1	Bile Acid Biosynthesis, Neutral Pathway	**cholesterol**	N/A	N/A	N/A	N/A	N/A	1.33	1.25	1.10	1.12	-1.37
M1	Bile Acid Biosynthesis, Neutral Pathway	**glycocholic acid**	N/A	N/A	N/A	N/A	N/A	1.33	1.14	1.57	-2.50	1.33
M1	Bile Acid Biosynthesis, Neutral Pathway	**taurocholic acid**	N/A	N/A	N/A	N/A	N/A	-1.37	-1.73	-1.23	-1.33	1.87
M1	Bile Acid Biosynthesis, Neutral Pathway	**taurine**	N/A	N/A	N/A	N/A	N/A	-1.10	-1.01	-1.19	-1.13	-1.24
M1	Bile Acid Biosynthesis, Neutral Pathway	**NAD+**	N/A	N/A	N/A	N/A	N/A	-1.50	-1.31	-1.15	-1.51	-1.42
M1	Bile Acid Biosynthesis, Neutral Pathway	CYP7A1	2.68	3.06	1.45	1.67	2.27	N/A	N/A	N/A	N/A	N/A
M1	Bile Acid Biosynthesis, Neutral Pathway	SLC27A5	-1.02	-1.15	-1.33	-1.63	-1.49	N/A	N/A	N/A	N/A	N/A
M2	Purine Ribonucleosides Degradation to Ribose-1-phosphate	**adenine**	N/A	N/A	N/A	N/A	N/A	1.17	1.24	1.67	1.61	1.96
M2	Purine Ribonucleosides Degradation to Ribose-1-phosphate	**guanine**	N/A	N/A	N/A	N/A	N/A	1.23	1.29	1.54	2.24	1.32
M2	Purine Ribonucleosides Degradation to Ribose-1-phosphate	**hypoxanthine**	N/A	N/A	N/A	N/A	N/A	1.50	1.51	1.29	1.55	1.38
M2	Purine Ribonucleosides Degradation to Ribose-1-phosphate	**inosine**	N/A	N/A	N/A	N/A	N/A	1.46	1.35	1.60	1.32	1.32
M2	Purine Ribonucleosides Degradation to Ribose-1-phosphate	**D-ribose-5-phosphate**	N/A	N/A	N/A	N/A	N/A	1.47	1.62	1.16	1.03	-1.08
M2	Purine Ribonucleosides Degradation to Ribose-1-phosphate	**guanosine**	N/A	N/A	N/A	N/A	N/A	1.13	1.33	1.34	-1.64	1.43
M2	Purine Ribonucleosides Degradation to Ribose-1-phosphate	**adenosine**	N/A	N/A	N/A	N/A	N/A	-1.30	-1.22	1.72	-2.22	-1.94
M3	Histamine Degradation	**imidazole-4-acetic acid**	N/A	N/A	N/A	N/A	N/A	1.47	2.72	1.28	1.93	4.22
M3	Histamine Degradation	**S-adenosylhomocysteine**	N/A	N/A	N/A	N/A	N/A	1.08	1.07	1.22	1.09	1.20
M3	Histamine Degradation	**histamine**	N/A	N/A	N/A	N/A	N/A	-1.22	1.10	-1.63	1.05	-1.17
M3	Histamine Degradation	**L-glutamic acid**	N/A	N/A	N/A	N/A	N/A	-1.09	-1.79	-1.06	-1.40	1.20
M3	Histamine Degradation	**NAD+**	N/A	N/A	N/A	N/A	N/A	-1.50	-1.31	-1.15	-1.51	-1.42
M3	Histamine Degradation	Aldh1a7	-1.82	4.23	8.60	9.54	69.36	N/A	N/A	N/A	N/A	N/A
M3	Histamine Degradation	ALDH1A1	1.16	1.53	1.85	2.58	2.84	N/A	N/A	N/A	N/A	N/A
M3	Histamine Degradation	ALDH1A3	1.18	1.10	-1.09	-1.59	-1.82	N/A	N/A	N/A	N/A	N/A
T1	Xenobiotic Metabolism CAR Signaling Pathway	CYP2B6	3.09	5.45	6.93	9.00	10.51	N/A	N/A	N/A	N/A	N/A
T1	Xenobiotic Metabolism CAR Signaling Pathway	ABCC3	1.33	2.80	5.63	9.82	12.71	N/A	N/A	N/A	N/A	N/A
T1	Xenobiotic Metabolism CAR Signaling Pathway	GSTA3	1.05	1.44	2.46	4.63	6.59	N/A	N/A	N/A	N/A	N/A
T1	Xenobiotic Metabolism CAR Signaling Pathway	CYP1A1	1.23	1.24	1.61	3.04	6.07	N/A	N/A	N/A	N/A	N/A
T1	Xenobiotic Metabolism CAR Signaling Pathway	ABCB1	1.60	2.02	2.09	2.80	4.37	N/A	N/A	N/A	N/A	N/A
T1	Xenobiotic Metabolism CAR Signaling Pathway	UGT2B17	1.51	1.75	2.27	2.64	3.04	N/A	N/A	N/A	N/A	N/A
T1	Xenobiotic Metabolism CAR Signaling Pathway	ALDH1A1	1.16	1.53	1.85	2.58	2.84	N/A	N/A	N/A	N/A	N/A
T1	Xenobiotic Metabolism CAR Signaling Pathway	UGT2B11	1.21	1.42	1.67	2.21	2.84	N/A	N/A	N/A	N/A	N/A
T1	Xenobiotic Metabolism CAR Signaling Pathway	UGT2B4	1.18	1.22	1.50	2.20	2.33	N/A	N/A	N/A	N/A	N/A
T1	Xenobiotic Metabolism CAR Signaling Pathway	CYP3A5	1.21	1.28	1.57	2.05	2.27	N/A	N/A	N/A	N/A	N/A
T1	Xenobiotic Metabolism CAR Signaling Pathway	GSTA1	1.15	1.27	1.41	1.88	2.08	N/A	N/A	N/A	N/A	N/A
T1	Xenobiotic Metabolism CAR Signaling Pathway	MAP2K3	1.19	1.43	1.25	1.67	1.99	N/A	N/A	N/A	N/A	N/A
T1	Xenobiotic Metabolism CAR Signaling Pathway	UGT2B10	1.08	1.25	1.21	1.54	2.18	N/A	N/A	N/A	N/A	N/A
T1	Xenobiotic Metabolism CAR Signaling Pathway	MGST2	1.34	1.32	1.33	1.57	1.50	N/A	N/A	N/A	N/A	N/A
T1	Xenobiotic Metabolism CAR Signaling Pathway	PPP2R1B	1.27	1.38	1.26	1.26	1.55	N/A	N/A	N/A	N/A	N/A
T1	Xenobiotic Metabolism CAR Signaling Pathway	PPP2R3A	1.16	1.16	1.40	1.57	1.32	N/A	N/A	N/A	N/A	N/A
T1	Xenobiotic Metabolism CAR Signaling Pathway	GSTP1	-1.09	-1.16	1.18	2.94	4.64	N/A	N/A	N/A	N/A	N/A
T1	Xenobiotic Metabolism CAR Signaling Pathway	ABCC2	1.12	1.04	1.15	1.27	1.58	N/A	N/A	N/A	N/A	N/A
T1	Xenobiotic Metabolism CAR Signaling Pathway	SULT2B1	1.04	1.02	1.05	1.43	1.60	N/A	N/A	N/A	N/A	N/A
T1	Xenobiotic Metabolism CAR Signaling Pathway	SULT2A1	-1.16	1.07	1.22	1.32	2.28	N/A	N/A	N/A	N/A	N/A
T1	Xenobiotic Metabolism CAR Signaling Pathway	FMO1	1.29	1.17	-1.03	-1.23	-1.81	N/A	N/A	N/A	N/A	N/A
T1	Xenobiotic Metabolism CAR Signaling Pathway	ALDH1A3	1.18	1.10	-1.09	-1.59	-1.82	N/A	N/A	N/A	N/A	N/A
T1	Xenobiotic Metabolism CAR Signaling Pathway	SULT1C3	1.02	1.03	-1.06	-1.24	-2.12	N/A	N/A	N/A	N/A	N/A
T1	Xenobiotic Metabolism CAR Signaling Pathway	PPP2R2B	-1.05	1.33	1.02	-1.59	-2.40	N/A	N/A	N/A	N/A	N/A
T1	Xenobiotic Metabolism CAR Signaling Pathway	SCAND1	1.06	-1.08	-1.09	-1.18	-1.54	N/A	N/A	N/A	N/A	N/A
T1	Xenobiotic Metabolism CAR Signaling Pathway	Sult1c2 (includes others)	-1.04	1.05	-1.19	-1.38	-1.71	N/A	N/A	N/A	N/A	N/A
T1	Xenobiotic Metabolism CAR Signaling Pathway	CYP1A2	-1.04	-1.10	-1.41	-1.67	-1.56	N/A	N/A	N/A	N/A	N/A
T1	Xenobiotic Metabolism CAR Signaling Pathway	EGFR	-2.01	-2.21	-2.15	-1.63	-1.90	N/A	N/A	N/A	N/A	N/A
T2	Xenobiotic Metabolism General Signaling Pathway	GSTA3	1.05	1.44	2.46	4.63	6.59	N/A	N/A	N/A	N/A	N/A
T2	Xenobiotic Metabolism General Signaling Pathway	UGT2B17	1.51	1.75	2.27	2.64	3.04	N/A	N/A	N/A	N/A	N/A
T2	Xenobiotic Metabolism General Signaling Pathway	NQO1	1.11	1.64	1.75	2.64	3.45	N/A	N/A	N/A	N/A	N/A
T2	Xenobiotic Metabolism General Signaling Pathway	UGT2B11	1.21	1.42	1.67	2.21	2.84	N/A	N/A	N/A	N/A	N/A
T2	Xenobiotic Metabolism General Signaling Pathway	HMOX1	1.11	1.40	1.28	2.03	3.35	N/A	N/A	N/A	N/A	N/A
T2	Xenobiotic Metabolism General Signaling Pathway	UGT2B4	1.18	1.22	1.50	2.20	2.33	N/A	N/A	N/A	N/A	N/A
T2	Xenobiotic Metabolism General Signaling Pathway	GSTA1	1.15	1.27	1.41	1.88	2.08	N/A	N/A	N/A	N/A	N/A
T2	Xenobiotic Metabolism General Signaling Pathway	MAP2K3	1.19	1.43	1.25	1.67	1.99	N/A	N/A	N/A	N/A	N/A
T2	Xenobiotic Metabolism General Signaling Pathway	UGT2B10	1.08	1.25	1.21	1.54	2.18	N/A	N/A	N/A	N/A	N/A
T2	Xenobiotic Metabolism General Signaling Pathway	MGST2	1.34	1.32	1.33	1.57	1.50	N/A	N/A	N/A	N/A	N/A
T2	Xenobiotic Metabolism General Signaling Pathway	GSTP1	-1.09	-1.16	1.18	2.94	4.64	N/A	N/A	N/A	N/A	N/A
T2	Xenobiotic Metabolism General Signaling Pathway	GCLC	-1.13	-1.13	1.04	1.25	2.11	N/A	N/A	N/A	N/A	N/A
T2	Xenobiotic Metabolism General Signaling Pathway	PIK3C2G	1.16	1.04	1.05	-1.04	-1.58	N/A	N/A	N/A	N/A	N/A
T3	Superpathway of Cholesterol Biosynthesis	HMGCR	1.18	1.33	1.94	2.96	2.23	N/A	N/A	N/A	N/A	N/A
T3	Superpathway of Cholesterol Biosynthesis	MSMO1	1.25	1.40	1.53	2.07	2.15	N/A	N/A	N/A	N/A	N/A
T3	Superpathway of Cholesterol Biosynthesis	MVD	1.01	1.24	1.41	2.26	2.26	N/A	N/A	N/A	N/A	N/A
T3	Superpathway of Cholesterol Biosynthesis	HSD17B7	1.15	1.35	1.37	1.84	1.76	N/A	N/A	N/A	N/A	N/A
T3	Superpathway of Cholesterol Biosynthesis	SQLE	1.07	1.13	1.31	1.78	1.78	N/A	N/A	N/A	N/A	N/A
T3	Superpathway of Cholesterol Biosynthesis	FDFT1	1.13	1.16	1.25	1.54	1.68	N/A	N/A	N/A	N/A	N/A
T3	Superpathway of Cholesterol Biosynthesis	HMGCS1	1.03	1.08	1.27	1.52	1.55	N/A	N/A	N/A	N/A	N/A
T3	Superpathway of Cholesterol Biosynthesis	SC5D	1.01	1.02	1.01	1.33	1.57	N/A	N/A	N/A	N/A	N/A
T3	Superpathway of Cholesterol Biosynthesis	LSS	-1.00	1.09	1.15	1.69	2.08	N/A	N/A	N/A	N/A	N/A
T3	Superpathway of Cholesterol Biosynthesis	IDI1	-1.12	1.26	1.18	1.63	1.99	N/A	N/A	N/A	N/A	N/A
T3	Superpathway of Cholesterol Biosynthesis	**cholesterol**	N/A	N/A	N/A	N/A	N/A	1.33	1.25	1.10	1.12	-1.37
T3	Superpathway of Cholesterol Biosynthesis	ACAT2	-1.12	-1.01	1.01	1.60	2.15	N/A	N/A	N/A	N/A	N/A
T3	Superpathway of Cholesterol Biosynthesis	CYP51A1	-1.06	-1.03	1.08	1.29	1.53	N/A	N/A	N/A	N/A	N/A

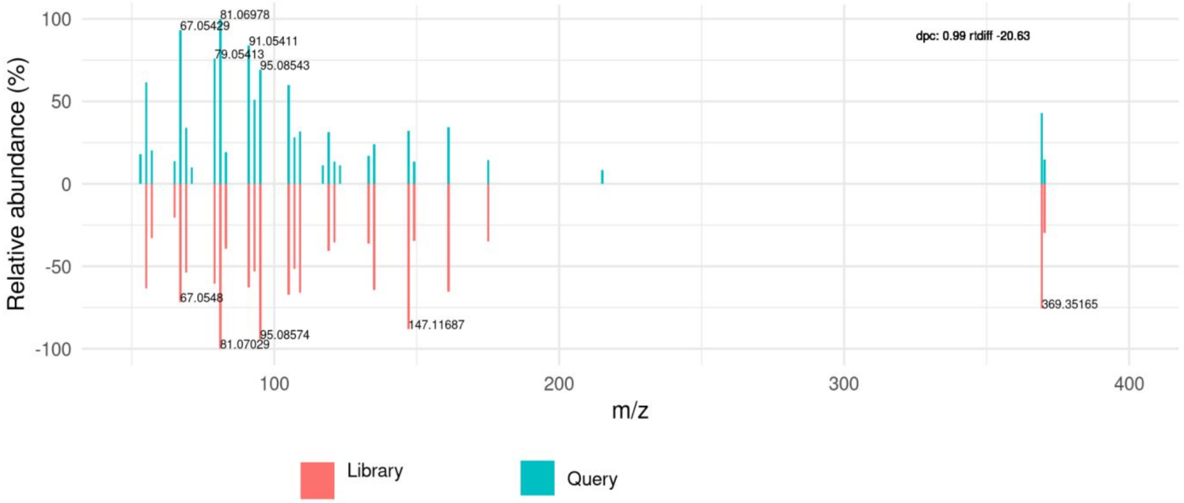
dpc: 0.99 rtdiff -20.63
Relative abundance (%)
m/z
81.06978
67.05429
91.05411
79.05413
95.08543
67.0548
95.08574
81.07029
147.11687
369.35165
Library
Query

a)

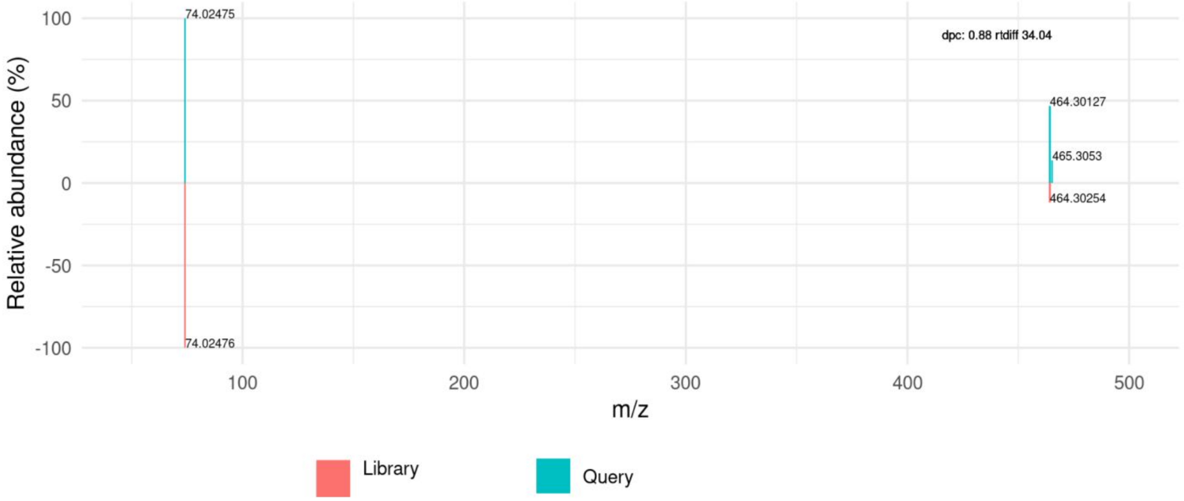
dpc: 0.88 rtdiff 34.04
Relative abundance (%)
m/z
74.02475
74.02476
464.30127
465.3053
464.30254
Library
Query

b)

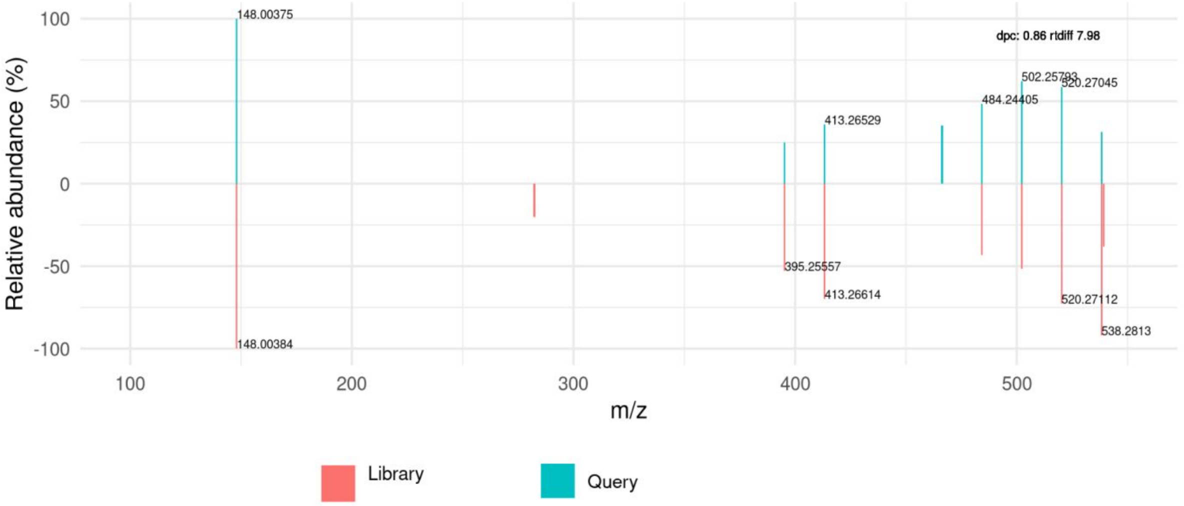
dpc: 0.86 rtdiff 7.98
Relative abundance (%)
m/z
148.00375
148.00384
413.26529
502.25793
520.27045
484.24405
395.25557
413.26614
520.27112
538.2813
Library
Query

c)

Figure S4. Spectral matching for selected metabolic features - a) cholesterol, b) glycocholic acid, c) taurocholic acid measured in the rat liver metabolome ("query") and also as analytical standards ("library"), allowing Metabolomics Standards Initiative level 1 identifications.

Table S3. Number of features measured in the 4 UHPLC-MS assays that were annotated - with varying levels of confidence - as parent TPhP or its biotransformation products, across different stages of data processing.

**One peak had a high number of missing values and was excluded from further analysis.*

Assay	Total number of tentatively annotated xenobiotic-related features	Total number of annotated xenobiotic-related features	Total number of representative annotated xenobiotic-related features
HILIC positive	144	25	6
HILIC negative	90	11	3
LIPIDS positive	114	9	2
LIPIDS negative	37	8*	1
Total	385	53*	12

Table S4. Summary of UHPLC-MS metabolomics measurements of TPhP and its biotransformation products, measured in rat liver.

Compound	Molecular formula	Assay	Ion form	m/z	RT (s)	MS^2	Number of TPhP signature MS^2 fragments	Selected as a representative feature?
TPhP	$C_{18}H_{15}O_4P$	LIPIDS positive	$[M+H]^+$	327.07768	77.6	Yes	9 (*m/z* 50.0155; 51.0233; 53.0389; 77.0385; 95.0490; 105.0446; 152.0619; 153.0692; 233.0358)	Yes
			$[M+NH_4]^+$	344.10425	77.6	Yes	2 (*m/z* 95.0490; 152.0620)	No
			$[M+Na]^+$	349.05960	77.6	No	N/A	No
DPhP	$C_{12}H_{11}O_4P$	HILIC negative	$[M-H]^-$	249.03206	41.8	Yes	5 (*m/z* 62.9640; 65.0396; 78.9590; 93.0345; 94.0379)	Yes
		HILIC positive	$[M+H]^+$	251.04674	41.6	Yes	10 (*m/z* 50.0156; 51.0234; 53.0390; 77.0385; 95.0491; 105.0448; 152.0621; 153.0700; 233.0362; 251.0468)	No
			$[M+NH_4]^+$	268.07340	41.6	No	N/A	No
		LIPIDS negative	$[M-H]^-$	249.03170	51.9	Yes	5 (*m/z* 62.9640; 65.0396; 78.9590; 93.0345; 94.0378)	No
M2	$C_{24}H_{23}O_{12}P$	HILIC negative	$[M+Na-2H]^-$	555.06685	275.2	Yes	1 (*m/z* 93.0345)	No
			$[M+Cl]^-$	569.06227	278.5	Yes	1 (*m/z* 93.0345)	No
			$[M+TFA-H]^-$	647.07896	275.7	Yes	1 (*m/z* 93.0345)	No
		HILIC positive	[M+H]+	535.09979	275.2	Yes	8 (*m/z* 51.0234; 53.0389;	Yes

							77.0386; 95.0492; 105.0448; 152.0621; 233.0364; 251.0470)	
			$[M+NH_4]^+$	552.12632	275.2	Yes	4 (*m/z* 95.0490; 152.0621; 233.0358; 251.0466)	No
			$[M+K]^+$	573.05554	275.2	No	N/A	No
			$[M+Na]^+$	557.08161	275.2	Yes	0	No
		LIPIDS negative	$[M-H]^-$	533.08552	41.7	No	N/A	No
M3	$C_{24}H_{23}O_{11}P$	HILIC negative	$[M-H]^-$	517.09033	250.4	Yes	4 (*m/z* 65.0395; 78.959; 93.0346; 94.0379)	Yes
		LIPIDS negative	$[M-H]^-$	517.09041	42.5	No	N/A	No
M4	$C_{18}H_{15}O_9PS$	HILIC negative	$[M-H]^-$	437.01002	25.7	Yes	5 (*m/z* 62.9638; 65.0396; 78.9590; 93.0345; 94.0379)	Yes
		HILIC positive HILIC positive HILIC positive	$[M+H]^+$	439.02401	25.7	Yes	10 (*m/z* 50.0156; 51.0234; 53.0390; 77.0384; 95.0492; 105.0448; 152.0620; 153.0702; 233.0364; 251.0468)	No
			$[M+NH_4]^+$	456.05098	25.7	Yes	10 (*m/z* 50.0156; 51.0234; 53.0390; 77.0385; 95.0491; 105.0448; 152.0620; 153.0699; 233.0360; 251.0465)	No
			$[M+Na]^+$	461.00629	25.6	No	N/A	No
		LIPIDS negative	$[M-H]^-$	437.01019	43.6	No	N/A	No
M5	$C_{18}H_{15}O_8PS$	HILIC negative	$[M-H]^-$	421.01536	26.1	Yes	5 (*m/z* 62.9640; 65.0397;	Yes

							78.9591; 93.0346; 94.0379)	
		HILIC positive	$[M+H]^+$	423.02913	26.1	Yes	7 (*m/z* 51.0234; 77.0384; 95.0492; 105.0448; 152.0621; 233.0362; 251.0466)	No
			$[M+NH_4]^+$	440.05610	26.1	Yes	8 (*m/z* 51.0234; 53.0390; 77.0384; 95.0491; 105.0448; 152.0620; 233.0361; 251.0467)	No
			$[M+Na]^+$	445.01136	26.0	No	N/A	No
		LIPIDS negative	$[M-H]^-$	421.01537	43.6	No	N/A	No
M6	$C_{18}H_{15}O_5P$	HILIC positive	$[M+H]^+$	343.07299	30.0	Yes	3 (*m/z* 51.; 95.0491; 152.0620)	Yes
			$[M+NH_4]^+$	360.09963	29.9	No	N/A	No
M7	$C_{18}H_{15}O_6P$	HILIC negative	$[M-H]^-$	357.05339	31.3	No	N/A	No
			$[M+Cl]^-$	393.03017	31.2	No	N/A	No
		HILIC positive	$[M+H]^+$	359.06771	31.2	No	N/A	No
			$[M+NH_4]^+$	376.09440	31.2	No	N/A	No
		LIPIDS negative	$[M-H]^-$	357.05347	51.9	Yes	4 (*m/z* 65.0395; 78.9590; 93.0345; 94.0379)	Yes
			$[M+TFA-H]^-$	471.04627	52.1	No	N/A	No
		LIPIDS positive	$[M+H]^+$	359.06747	51.9	Yes	4 (*m/z* 95.0490; 105.0446; 152.0619, 251.0455)	No
			$[M+NH_4]^+$	376.09405	51.9	No	N/A	No
			$[M+Na]^+$	381.04940	51.9	No	N/A	No
			$[M+ACN+H]^+$	390.10950	51.9	No	N/A	No
M8	$C_{19}H_{17}O_6P$	HILIC positive	$[M+H]^+$	373.08354	29.8	Yes	3 (*m/z* 51.0234; 95.0494; |233.0360)	No

			$[M+NH_4]^+$	390.11008	30.0	No	N/A	No
		LIPIDS positive	$[M+H]^+$	373.08323	57.3	Yes	5 (*m/z* 50.0159; 51.0237; 53.0393; 95.0494; 233.0358)	Yes
			$[M+Na]^+$	395.06519	57.4	No	N/A	No
M9	$C_{30}H_{31}O_{18}P$	HILIC negative	$[M-H]^-$	709.11815	415.0	Yes	1 (*m/z* 93.0345)	No
		HILIC positive	$[M+NH_4]^+$	728.15760	415.2	Yes	5 (*m/z* 95.0492; 152.0618; 153.0705; 233.0366; 251.0467)	Yes
			$[M+Na]^+$	733.11250	415.2	Yes	0	No
M10	$C_{25}H_{25}O_{12}P$	HILIC negative	$[M-H]^-$	547.10121	266.1	Yes	3 (*m/z* 78.9589; 93.0346; 94.0378)	No
		HILIC positive	$[M+NH_4]^+$	566.14198	265.9	Yes	4 (*m/z* 95.0491; 105.0446; 152.0619; 233.0361)	Yes
			$[M+K]^+$	587.07121	266.1	No	N/A	No
		LIPIDS negative	$[M-H]^-$	547.10094	42.1	No	N/A	No
M11	Unknown	HILIC positive	$[M+H]^+$	678.15090	367.8	Yes	3 (*m/z* 53.0389; 95.0492; 233.0363)	Yes
			$[M+Na]^+$	700.13283	368.0	No	N/A	No
			$[M+K]^+$	716.10659	367.8	No	N/A	No

a)

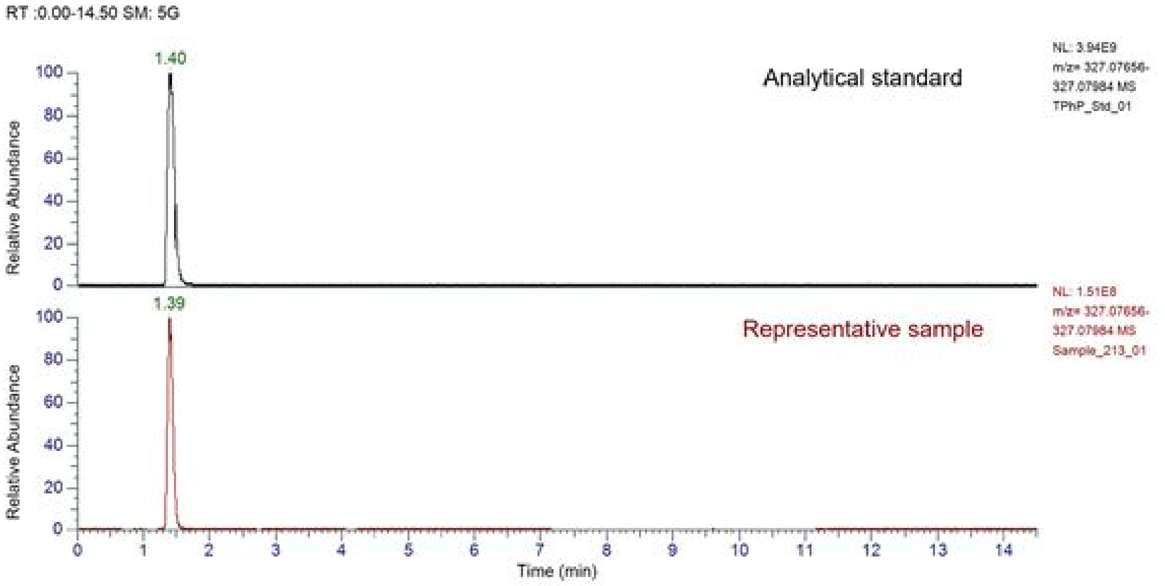
RT :0.00-14.50 SM: 5G
1.40
Analytical standard
NL: 3.94E9
m/z= 327.07656-
327.07984 MS
TPhP_Std_01
Relative Abundance
1.39
Representative sample
NL: 1.51E8
m/z= 327.07656-
327.07984 MS
Sample_213_01
Relative Abundance
Time (min)

b)

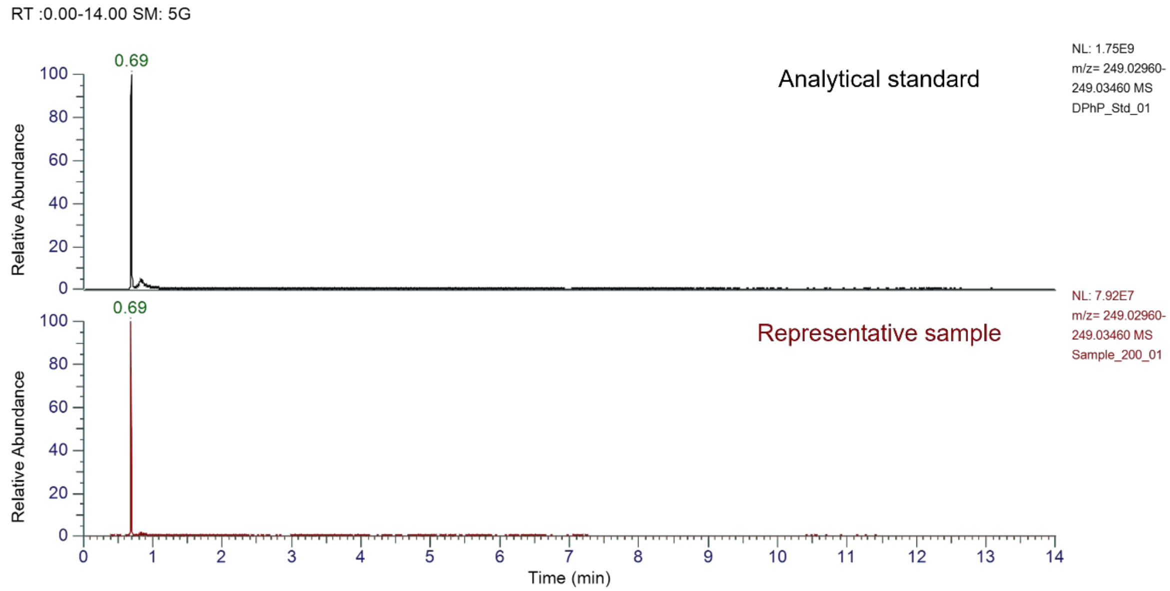
RT :0.00-14.00 SM: 5G
0.69
Analytical standard
NL: 1.75E9
m/z= 249.02960-
249.03460 MS
DPhP_Std_01
Relative Abundance
0.69
Representative sample
NL: 7.92E7
m/z= 249.02960-
249.03460 MS
Sample_200_01
Relative Abundance
Time (min)

c)

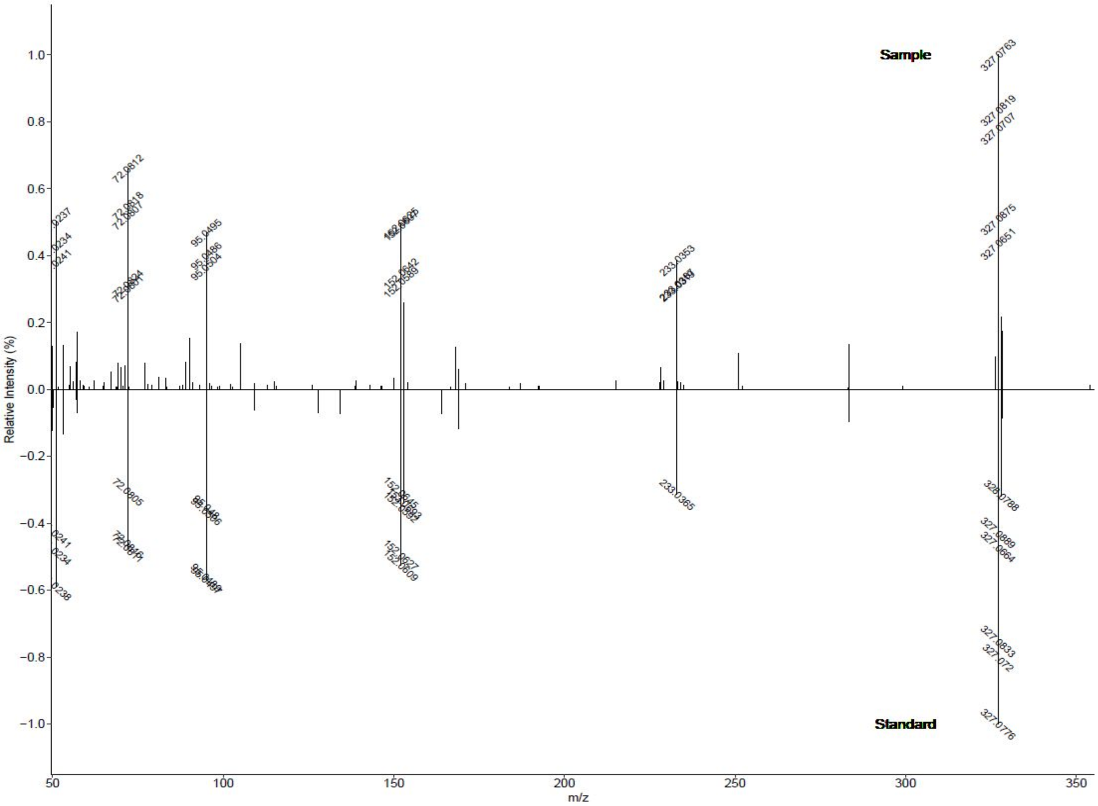

Sample
Standard
Relative Intensity (%)
m/z
327.0763
327.0819
327.0707
327.0675
327.0651
72.9812
95.0495
233.0353
233.0365
72.0905
327.0776

d)

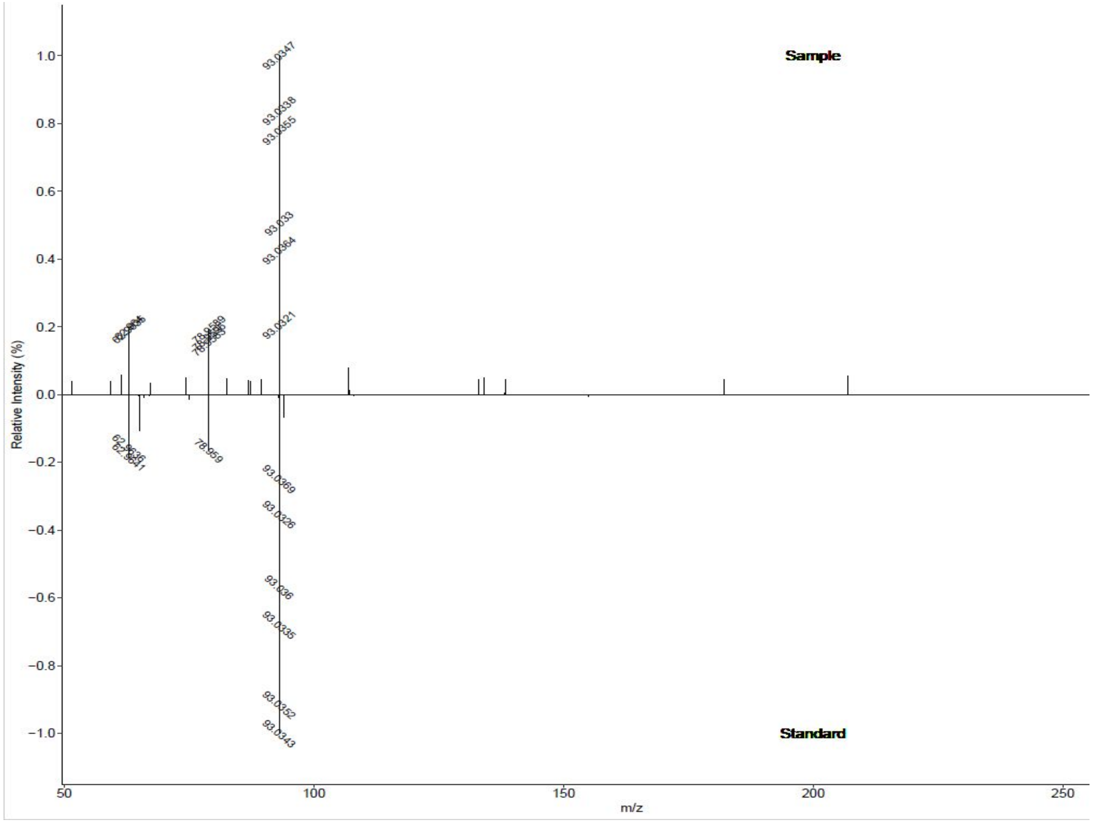

Sample
Standard
Relative Intensity (%)
m/z
93.0347
93.0338
93.0355
93.033
93.0364
93.0321
78.959
93.0369
93.0326
93.036
93.0335
93.0352
93.0343

Figure S5. Confirmation of MSI level 1 identification of (a and c) TPhP and (b and d) its primary biotransformation product DPhP by comparison to analytical standards.

Table S5. Summary of Spearman correlation analysis of the relative intensities of 12 representative features (for TPhP, DPhP and 10 other BTPs) with the levels of gene expression reported by the NTP.

Compound	Assay	Ion adduct	Positively correlated genes	Positively correlated significant genes	Negatively correlated genes	Negatively correlated significant genes
TPhP	LIPIDS positive	[M+H]+	901	561	533	360
DPHP	LIPIDS negative	[M-H]-	924	688	511	387
M2	HILIC positive	[M+H]+	909	678	527	401
M3	HILIC negative	[M-H]-	920	687	516	399
M4	LIPIDS negative	[M-H]-	907	461	529	300
M5	HILIC positive	[M+H]+	910	410	526	249
M6	HILIC positive	[M+H]+	910	671	526	369
M7	HILIC positive	[M+H]+	910	717	526	403
M8	LIPIDS positive	[M+H]+	911	692	524	400
M9	HILIC negative	[M-H]-	911	671	524	386
M10	HILIC negative	[M-H]-	916	673	519	395
M11	HILIC positive	[M+H]+	919	658	517	393
